# Supplementary material for: Waist, waist-height-ratio vs body mass index and the risks of multiple diseases: a cohort study with replication
Source: medRxiv. 2025 Oct 19:2025.10.16.25338152. Preprint. [Version 1] doi: 10.1101/2025.10.16.25338152 (PMC12633129; doi:10.1101/2025.10.16.25338152)

Waist, waist-height-ratio vs body mass index and the risks of multiple diseases: a cohort study with replication

Frederick Ho, PhD, Jaana Pentti, MSc

Prof. Mika Kivimäki, FMedSci, Prof. Naveed Sattar, FMedSci

SUPPLEMENT

Supplement eTable 1. Cut-points used to define overweight and obesity

	Female			Male		
	**BMI=25**	**BMI=30**	**BMI=35**	**BMI=25**	**BMI=30**	**BMI=35**
Historic						
WC (Lean et al, 1995)	80	88	88	94	102	102
Contemporary (2005-2019)						
WC	83	96	96	91	106	106
WHtR	0.52	0.60	0.60	0.52	0.61	0.61
UK Biobank (2007-2010)						
WC	80	93	104	89	103	116
WHtR	0.49	0.57	0.64	0.51	0.59	0.66
Whitehall (1991-1994)						
WC	75	88	100	88	103	115
WHtR	0.47	0.55	0.62	0.50	0.58	0.66

Supplement eTable 2. Associations of obesity with outcomes

		BMI				WC (UKB)				WHtR (UKB)			
		n	event	HR (95% CI)	P-value	n	event	HR (95% CI)	P-value	n	event	HR (95% CI)	P-value
16	Diabetes mellitus	118336	21367	6.79 (6.56-7.03)	< 0.0001	124369	23014	7.87 (7.58-8.18)	< 0.0001	120413	23122	8.87 (8.53-9.22)	< 0.0001
29	Sleep disorders	120522	5807	6.25 (5.85-6.67)	< 0.0001	126794	5939	6.38 (5.95-6.84)	< 0.0001	122981	5757	6.47 (6.05-6.92)	< 0.0001
60	Gout	121484	4924	4.60 (4.31-4.92)	< 0.0001	127814	5253	4.57 (4.25-4.92)	< 0.0001	123939	5056	4.65 (4.33-4.99)	< 0.0001
0.3	**Complex multimorbidity (4th disease)**	119945	32402	3.19 (3.13-3.26)	< 0.0001	126143	35230	3.45 (3.38-3.53)	< 0.0001	122232	35389	3.60 (3.52-3.68)	< 0.0001
33	Hypertension	109779	47696	2.79 (2.75-2.84)	< 0.0001	115391	51327	2.86 (2.81-2.90)	< 0.0001	111328	51052	3.03 (2.98-3.08)	< 0.0001
15	**Endocrine diseases**	110339	52914	2.78 (2.74-2.82)	< 0.0001	116068	56059	2.83 (2.79-2.87)	< 0.0001	111939	55456	2.94 (2.90-2.99)	< 0.0001
0.2	**Third disease**	116534	44389	2.78 (2.73-2.83)	< 0.0001	122486	48022	2.93 (2.88-2.98)	< 0.0001	118472	47822	3.01 (2.96-3.07)	< 0.0001
53	Liver diseases	121278	6547	2.53 (2.42-2.64)	< 0.0001	127559	7099	2.86 (2.73-3.00)	< 0.0001	123652	7072	3.04 (2.90-3.19)	< 0.0001
39	Heart failure	121083	8054	2.45 (2.35-2.55)	< 0.0001	127378	8829	2.57 (2.46-2.69)	< 0.0001	123474	8882	2.57 (2.46-2.68)	< 0.0001
61	Osteoarthritis	114910	29546	2.40 (2.35-2.44)	< 0.0001	121029	30639	2.22 (2.18-2.27)	< 0.0001	117075	30438	2.20 (2.16-2.25)	< 0.0001
0.1	**Simple multimorbidity (2nd disease)**	108016	55569	2.38 (2.35-2.41)	< 0.0001	113373	59830	2.44 (2.41-2.48)	< 0.0001	109304	58913	2.49 (2.45-2.52)	< 0.0001
66	Renal failure	121403	16099	2.35 (2.28-2.41)	< 0.0001	127677	17679	2.46 (2.39-2.53)	< 0.0001	123792	17890	2.53 (2.46-2.60)	< 0.0001
35	*Angina pectoris*	118050	8715	2.24 (2.16-2.33)	< 0.0001	124112	9428	2.25 (2.17-2.34)	< 0.0001	120110	9562	2.54 (2.44-2.64)	< 0.0001
55	Pancreatitis	121540	1219	2.23 (2.02-2.46)	< 0.0001	127840	1328	2.62 (2.35-2.91)	< 0.0001	123967	1310	2.69 (2.42-2.99)	< 0.0001
37	Pulmonary embolism	121326	3365	2.19 (2.06-2.32)	< 0.0001	127607	3636	2.36 (2.21-2.51)	< 0.0001	123788	3496	2.08 (1.96-2.21)	< 0.0001
32	**Circulatory diseases**	101444	55033	2.07 (2.04-2.09)	< 0.0001	106542	59417	2.11 (2.08-2.14)	< 0.0001	102783	58454	2.15 (2.12-2.18)	< 0.0001
57	Eczema and skin infections	118722	9011	2.05 (1.98-2.12)	< 0.0001	124922	9774	2.18 (2.10-2.26)	< 0.0001	121123	9472	2.16 (2.09-2.24)	< 0.0001
34	Ischaemic heart disease	116327	16759	2.00 (1.95-2.05)	< 0.0001	122269	18304	2.03 (1.98-2.08)	< 0.0001	118200	18277	2.18 (2.12-2.24)	< 0.0001
45	Deep vein thrombosis	120942	2760	1.90 (1.79-2.02)	< 0.0001	127178	2941	1.98 (1.86-2.12)	< 0.0001	123377	2814	1.79 (1.68-1.91)	< 0.0001
62	Sciatica	120505	5107	1.86 (1.78-1.94)	< 0.0001	126797	5326	1.80 (1.72-1.88)	< 0.0001	123011	5266	1.78 (1.71-1.87)	< 0.0001
10	Kidney cancer	121866	765	1.85 (1.64-2.08)	< 0.0001	128206	837	1.95 (1.72-2.21)	< 0.0001	124334	820	1.91 (1.69-2.16)	< 0.0001
63	Backpain	119048	9101	1.81 (1.75-1.87)	< 0.0001	125263	9680	1.82 (1.76-1.88)	< 0.0001	121410	9665	1.86 (1.80-1.92)	< 0.0001
21	Mood disorders	120312	10227	1.79 (1.74-1.85)	< 0.0001	126549	10998	1.97 (1.91-2.04)	< 0.0001	122670	10879	2.01 (1.95-2.07)	< 0.0001
49	Asthma	117728	12513	1.79 (1.74-1.84)	< 0.0001	123727	13458	2.00 (1.94-2.06)	< 0.0001	119740	13468	2.05 (1.99-2.11)	< 0.0001
38	Arrhythmias	119804	15201	1.74 (1.69-1.78)	< 0.0001	125892	16855	1.82 (1.77-1.87)	< 0.0001	122132	16070	1.60 (1.56-1.64)	< 0.0001
64	Soft tissue diseases	115140	15702	1.73 (1.69-1.77)	< 0.0001	121211	16352	1.70 (1.66-1.74)	< 0.0001	117409	16157	1.72 (1.67-1.76)	< 0.0001
36	*Myocardial infarction*	120092	4555	1.73 (1.65-1.81)	< 0.0001	126284	4970	1.72 (1.64-1.81)	< 0.0001	122342	5021	1.94 (1.85-2.04)	< 0.0001
58	**Musculoskeletal diseases**	103567	45213	1.71 (1.69-1.73)	< 0.0001	109188	47931	1.67 (1.65-1.70)	< 0.0001	105506	47273	1.68 (1.65-1.70)	< 0.0001
23	**Neurological disorders**	114226	21683	1.69 (1.66-1.73)	< 0.0001	120293	23173	1.71 (1.68-1.75)	< 0.0001	116360	22972	1.77 (1.74-1.81)	< 0.0001
54	Alcohol-related liver diseases	121881	660	1.69 (1.49-1.91)	< 0.0001	128215	777	2.26 (1.96-2.60)	< 0.0001	124320	787	2.79 (2.42-3.23)	< 0.0001
67	**Circulatory and respiratory symptoms**	111022	21308	1.57 (1.54-1.60)	< 0.0001	116786	23051	1.64 (1.60-1.67)	< 0.0001	112907	22808	1.65 (1.62-1.69)	< 0.0001
65	**Genitourinary diseases**	101913	32241	1.54 (1.51-1.57)	< 0.0001	107517	35095	1.63 (1.61-1.66)	< 0.0001	103469	34544	1.64 (1.61-1.67)	< 0.0001
2	Bacterial infections	120550	12966	1.51 (1.47-1.56)	< 0.0001	126763	14169	1.65 (1.60-1.69)	< 0.0001	122856	14266	1.69 (1.65-1.74)	< 0.0001
47	Respiratory tract infections	120869	10996	1.51 (1.47-1.56)	< 0.0001	127102	12557	1.75 (1.69-1.80)	< 0.0001	123212	12637	1.81 (1.75-1.87)	< 0.0001
1	**Infectious diseases**	117400	20166	1.49 (1.46-1.53)	< 0.0001	123502	22005	1.60 (1.57-1.64)	< 0.0001	119511	21971	1.64 (1.61-1.68)	< 0.0001
14	Anaemia	119031	15010	1.49 (1.45-1.52)	< 0.0001	125205	16245	1.58 (1.54-1.62)	< 0.0001	121242	16550	1.67 (1.63-1.71)	< 0.0001
46	**Respiratory diseases**	112313	31280	1.48 (1.46-1.51)	< 0.0001	117977	34459	1.63 (1.60-1.66)	< 0.0001	114115	34334	1.67 (1.65-1.70)	< 0.0001
43	*Cerebral infarction*	121342	3218	1.48 (1.40-1.56)	< 0.0001	127645	3632	1.55 (1.46-1.64)	< 0.0001	123744	3649	1.59 (1.50-1.68)	< 0.0001
44	Arteriosclerosis	121812	1267	1.48 (1.36-1.62)	< 0.0001	128139	1489	1.63 (1.48-1.79)	< 0.0001	124236	1532	1.86 (1.69-2.05)	< 0.0001
70	**Poisoning**	121084	1219	1.48 (1.36-1.61)	< 0.0001	127377	1328	1.61 (1.48-1.76)	< 0.0001	123505	1305	1.70 (1.56-1.86)	< 0.0001
13	**Blood diseases**	118569	16756	1.45 (1.42-1.48)	< 0.0001	124679	18100	1.54 (1.50-1.58)	< 0.0001	120724	18356	1.61 (1.57-1.65)	< 0.0001
56	**Skin diseases**	113576	17798	1.44 (1.40-1.47)	< 0.0001	119471	19566	1.52 (1.48-1.55)	< 0.0001	115772	18951	1.47 (1.44-1.51)	< 0.0001
17	**Mental and behavioural disorders**	118719	23311	1.43 (1.40-1.45)	< 0.0001	124752	25711	1.54 (1.51-1.57)	< 0.0001	120832	25555	1.60 (1.57-1.63)	< 0.0001
22	Neurotic disorders	121215	7903	1.38 (1.33-1.42)	< 0.0001	127517	8603	1.52 (1.47-1.57)	< 0.0001	123626	8580	1.53 (1.48-1.59)	< 0.0001
28	Transient ischaemic attack	121553	1385	1.35 (1.25-1.46)	< 0.0001	127874	1561	1.37 (1.26-1.48)	< 0.0001	123966	1569	1.46 (1.35-1.59)	< 0.0001
50	**Digestive system diseases**	95804	47367	1.34 (1.32-1.36)	< 0.0001	100308	50807	1.41 (1.39-1.43)	< 0.0001	96609	49761	1.44 (1.42-1.45)	< 0.0001
20	Psychotic disorders	121749	516	1.34 (1.18-1.52)	0.003	128060	619	1.65 (1.44-1.88)	< 0.0001	124186	622	1.81 (1.58-2.07)	< 0.0001
40	Cerebrovascular diseases	120769	6744	1.33 (1.28-1.38)	< 0.0001	127039	7706	1.43 (1.38-1.49)	< 0.0001	123121	7824	1.50 (1.45-1.56)	< 0.0001
48	Chronic obstructive pulmonary disease	121080	7799	1.33 (1.28-1.37)	< 0.0001	127307	9265	1.57 (1.52-1.63)	< 0.0001	123376	9504	1.70 (1.64-1.76)	< 0.0001
0	**All-cause mortality**	122001	13702	1.31 (1.28-1.35)	< 0.0001	128358	15696	1.42 (1.38-1.46)	< 0.0001	124472	15872	1.47 (1.43-1.51)	< 0.0001
59	Rheumatoid arthritis	118415	18570	1.31 (1.28-1.34)	< 0.0001	124569	20238	1.36 (1.33-1.39)	< 0.0001	120554	20737	1.41 (1.38-1.44)	< 0.0001
41	Stroke	121101	3955	1.31 (1.25-1.37)	< 0.0001	127382	4490	1.38 (1.31-1.45)	< 0.0001	123482	4518	1.41 (1.35-1.48)	< 0.0001
19	Substance use disorders	120940	8876	1.30 (1.26-1.34)	< 0.0001	127123	10124	1.40 (1.36-1.45)	< 0.0001	123211	9973	1.49 (1.44-1.54)	< 0.0001
73	**Self-harm**	121309	625	1.30 (1.16-1.46)	0.002	127628	686	1.41 (1.26-1.59)	< 0.0001	123747	662	1.49 (1.33-1.67)	< 0.0001
30	**Eye diseases**	116484	23717	1.24 (1.21-1.26)	< 0.0001	122301	26375	1.28 (1.26-1.31)	< 0.0001	118330	26759	1.31 (1.29-1.34)	< 0.0001
26	Epilepsy	121440	1655	1.24 (1.15-1.33)	< 0.0001	127763	1856	1.37 (1.27-1.47)	< 0.0001	123867	1857	1.47 (1.36-1.58)	< 0.0001
3	Viral infections	121161	1863	1.22 (1.14-1.30)	< 0.0001	127479	1985	1.23 (1.15-1.31)	< 0.0001	123598	1989	1.26 (1.18-1.35)	< 0.0001
5	Colorectal cancer	121486	2309	1.21 (1.14-1.28)	< 0.0001	127739	2626	1.29 (1.21-1.37)	< 0.0001	123872	2545	1.24 (1.17-1.32)	< 0.0001
31	**Ear diseases**	121334	945	1.21 (1.10-1.33)	0.01	127682	1024	1.24 (1.13-1.36)	0.004	123782	1018	1.30 (1.18-1.43)	< 0.0001
68	**Digestive and abdominal symptoms**	111863	22042	1.20 (1.18-1.23)	< 0.0001	117703	23828	1.27 (1.24-1.29)	< 0.0001	113697	23712	1.29 (1.27-1.32)	< 0.0001
52	Inflammatory bowel disease	118489	5575	1.20 (1.15-1.24)	< 0.0001	124584	6146	1.31 (1.26-1.36)	< 0.0001	120646	6103	1.34 (1.29-1.39)	< 0.0001
8	Breast cancer (Female)	62535	3171	1.15 (1.10-1.21)	< 0.0001	64015	3322	1.22 (1.16-1.28)	< 0.0001	65942	3323	1.15 (1.09-1.20)	< 0.0001
27	Headaches	121470	2004	1.15 (1.08-1.23)	0.002	127811	2037	1.18 (1.11-1.25)	0.0001	123920	2006	1.15 (1.08-1.22)	0.006
72	**Fall**	118854	10306	1.12 (1.09-1.15)	< 0.0001	124919	11605	1.22 (1.18-1.25)	< 0.0001	121056	11586	1.20 (1.17-1.24)	< 0.0001
4	**Cancers**	116775	20966	1.07 (1.05-1.09)	< 0.0001	122554	23432	1.13 (1.10-1.15)	< 0.0001	118703	22644	1.08 (1.05-1.10)	< 0.0001
69	**Injury**	115233	14186	1.06 (1.04-1.09)	< 0.0001	121175	15702	1.13 (1.11-1.16)	< 0.0001	117503	15372	1.12 (1.09-1.14)	< 0.0001
9	Prostate cancer (Male)	57328	3135	0.92 (0.88-0.97)	0.02	61993	3728	0.95 (0.91-1.01)	1.00	56135	3374	0.90 (0.85-0.94)	0.0007
7	Melanoma	120796	5370	0.85 (0.82-0.88)	< 0.0001	126999	6116	0.89 (0.86-0.92)	< 0.0001	123122	5806	0.83 (0.80-0.86)	< 0.0001
71	**Road accidents**	121046	1279	0.74 (0.69-0.80)	< 0.0001	127382	1360	0.74 (0.69-0.79)	< 0.0001	123535	1266	0.71 (0.66-0.76)	< 0.0001

Supplement eTable 3. Associations of altneriave definitions of obesity with outcomes

		BMI				WC (historic)				WC (HES-SHS)				WHtR (HES-SHS)			
		n	event	HR (95% CI)	P-value	n	event	HR (95% CI)	P-value	n	event	HR (95% CI)	P-value	n	event	HR (95% CI)	P-value
16	Diabetes mellitus	118336	21367	6.79 (6.56-7.03)	< 0.0001	163798	26052	5.60 (5.43-5.77)	< 0.0001	92188	19215	7.92 (7.67-8.19)	< 0.0001	79920	18262	9.00 (8.71-9.30)	< 0.0001
29	Sleep disorders	120522	5807	6.25 (5.85-6.67)	< 0.0001	166542	6468	4.91 (4.64-5.20)	< 0.0001	94299	5115	6.83 (6.43-7.27)	< 0.0001	82011	4791	7.14 (6.73-7.57)	< 0.0001
60	Gout	121484	4924	4.60 (4.31-4.92)	< 0.0001	167646	5779	3.23 (3.06-3.41)	< 0.0001	95161	4238	4.48 (4.21-4.78)	< 0.0001	82787	3896	4.73 (4.44-5.04)	< 0.0001
0.3	**Complex multimorbidity (4th disease)**	119945	32402	3.19 (3.13-3.26)	< 0.0001	165793	41485	2.84 (2.79-2.89)	< 0.0001	93710	28530	3.57 (3.50-3.65)	< 0.0001	81352	26571	3.77 (3.70-3.85)	< 0.0001
33	Hypertension	109779	47696	2.79 (2.75-2.84)	< 0.0001	152872	62936	2.40 (2.37-2.43)	< 0.0001	85148	39856	2.88 (2.84-2.93)	< 0.0001	73296	36334	3.06 (3.02-3.11)	< 0.0001
15	**Endocrine diseases**	110339	52914	2.78 (2.74-2.82)	< 0.0001	153621	68717	2.42 (2.39-2.45)	< 0.0001	85596	43985	2.93 (2.89-2.97)	< 0.0001	73754	39929	3.10 (3.05-3.14)	< 0.0001
0.2	**Third disease**	116534	44389	2.78 (2.73-2.83)	< 0.0001	161593	58005	2.48 (2.44-2.52)	< 0.0001	90686	38062	3.03 (2.98-3.08)	< 0.0001	78482	34886	3.16 (3.11-3.21)	< 0.0001
53	Liver diseases	121278	6547	2.53 (2.42-2.64)	< 0.0001	167272	8451	2.49 (2.40-2.60)	< 0.0001	94961	5755	2.97 (2.84-3.11)	< 0.0001	82592	5298	3.09 (2.96-3.23)	< 0.0001
39	Heart failure	121083	8054	2.45 (2.35-2.55)	< 0.0001	167137	10103	2.24 (2.16-2.32)	< 0.0001	94778	7256	2.75 (2.64-2.86)	< 0.0001	82416	6928	2.89 (2.77-3.00)	< 0.0001
61	Osteoarthritis	114910	29546	2.40 (2.35-2.44)	< 0.0001	159263	38625	1.97 (1.94-2.00)	< 0.0001	89689	23969	2.26 (2.21-2.30)	< 0.0001	77783	21562	2.26 (2.21-2.30)	< 0.0001
0.1	**Simple multimorbidity (2nd disease)**	108016	55569	2.38 (2.35-2.41)	< 0.0001	150710	74413	2.12 (2.09-2.14)	< 0.0001	83394	46171	2.53 (2.49-2.56)	< 0.0001	71672	41429	2.59 (2.56-2.63)	< 0.0001
66	Renal failure	121403	16099	2.35 (2.28-2.41)	< 0.0001	167466	21111	2.15 (2.09-2.20)	< 0.0001	95066	14233	2.55 (2.48-2.62)	< 0.0001	82683	13432	2.68 (2.61-2.75)	< 0.0001
35	*Angina pectoris*	118050	8715	2.24 (2.16-2.33)	< 0.0001	163298	11227	1.92 (1.86-1.99)	< 0.0001	92280	7356	2.22 (2.14-2.31)	< 0.0001	79966	6994	2.48 (2.39-2.57)	< 0.0001
55	Pancreatitis	121540	1219	2.23 (2.02-2.46)	< 0.0001	167623	1604	2.31 (2.11-2.53)	< 0.0001	95206	1074	2.61 (2.37-2.88)	< 0.0001	82839	949	2.59 (2.34-2.86)	< 0.0001
37	Pulmonary embolism	121326	3365	2.19 (2.06-2.32)	< 0.0001	167361	4453	2.08 (1.97-2.19)	< 0.0001	95003	2880	2.33 (2.20-2.47)	< 0.0001	82692	2532	2.10 (1.98-2.22)	< 0.0001
32	**Circulatory diseases**	101444	55033	2.07 (2.04-2.09)	< 0.0001	141400	74344	1.87 (1.85-1.90)	< 0.0001	78591	45482	2.17 (2.14-2.20)	< 0.0001	67613	40693	2.23 (2.20-2.26)	< 0.0001
57	Eczema and skin infections	118722	9011	2.05 (1.98-2.12)	< 0.0001	164217	11576	1.98 (1.92-2.05)	< 0.0001	92770	7916	2.35 (2.27-2.44)	< 0.0001	80656	7186	2.38 (2.30-2.46)	< 0.0001
34	Ischaemic heart disease	116327	16759	2.00 (1.95-2.05)	< 0.0001	161135	21790	1.79 (1.75-1.83)	< 0.0001	90838	14211	2.05 (2.00-2.10)	< 0.0001	78576	13246	2.22 (2.16-2.28)	< 0.0001
45	Deep vein thrombosis	120942	2760	1.90 (1.79-2.02)	< 0.0001	166887	3586	1.77 (1.67-1.87)	< 0.0001	94641	2334	2.05 (1.93-2.18)	< 0.0001	82378	2027	1.82 (1.71-1.94)	< 0.0001
62	Sciatica	120505	5107	1.86 (1.78-1.94)	< 0.0001	166260	6690	1.64 (1.58-1.71)	< 0.0001	94403	4156	1.80 (1.73-1.89)	< 0.0001	82169	3658	1.77 (1.69-1.85)	< 0.0001
10	Kidney cancer	121866	765	1.85 (1.64-2.08)	< 0.0001	168052	1008	1.72 (1.55-1.90)	< 0.0001	95485	689	1.98 (1.76-2.23)	< 0.0001	83106	590	1.89 (1.67-2.13)	< 0.0001
63	Backpain	119048	9101	1.81 (1.75-1.87)	< 0.0001	164385	12147	1.69 (1.64-1.74)	< 0.0001	93158	7607	1.87 (1.81-1.93)	< 0.0001	81024	6913	1.90 (1.84-1.97)	< 0.0001
21	Mood disorders	120312	10227	1.79 (1.74-1.85)	< 0.0001	166025	13910	1.83 (1.78-1.88)	< 0.0001	94143	8801	2.04 (1.98-2.10)	< 0.0001	81873	7869	2.06 (1.99-2.12)	< 0.0001
49	Asthma	117728	12513	1.79 (1.74-1.84)	< 0.0001	162497	16913	1.77 (1.73-1.82)	< 0.0001	91868	10601	2.02 (1.97-2.08)	< 0.0001	79717	9659	2.12 (2.06-2.18)	< 0.0001
38	Arrhythmias	119804	15201	1.74 (1.69-1.78)	< 0.0001	165317	20102	1.70 (1.66-1.74)	< 0.0001	93665	13352	1.95 (1.90-2.00)	< 0.0001	81517	11725	1.75 (1.71-1.80)	< 0.0001
64	Soft tissue diseases	115140	15702	1.73 (1.69-1.77)	< 0.0001	159293	20608	1.55 (1.52-1.58)	< 0.0001	90011	12824	1.75 (1.71-1.80)	< 0.0001	78148	11478	1.77 (1.73-1.81)	< 0.0001
36	*Myocardial infarction*	120092	4555	1.73 (1.65-1.81)	< 0.0001	165880	5916	1.55 (1.49-1.62)	< 0.0001	94005	3800	1.72 (1.64-1.80)	< 0.0001	81643	3558	1.90 (1.81-1.99)	< 0.0001
58	**Musculoskeletal diseases**	103567	45213	1.71 (1.69-1.73)	< 0.0001	144004	61376	1.55 (1.53-1.57)	< 0.0001	80705	36857	1.73 (1.70-1.75)	< 0.0001	69827	32751	1.74 (1.72-1.77)	< 0.0001
23	**Neurological disorders**	114226	21683	1.69 (1.66-1.73)	< 0.0001	158266	28624	1.59 (1.56-1.62)	< 0.0001	89148	18252	1.81 (1.77-1.85)	< 0.0001	77210	16543	1.88 (1.84-1.92)	< 0.0001
54	Alcohol-related liver diseases	121881	660	1.69 (1.49-1.91)	< 0.0001	168065	874	2.18 (1.94-2.46)	< 0.0001	95498	628	2.52 (2.20-2.89)	< 0.0001	83094	594	2.94 (2.57-3.38)	< 0.0001
67	**Circulatory and respiratory symptoms**	111022	21308	1.57 (1.54-1.60)	< 0.0001	154074	28864	1.52 (1.50-1.55)	< 0.0001	86510	17738	1.66 (1.63-1.70)	< 0.0001	74884	16067	1.71 (1.67-1.74)	< 0.0001
65	**Genitourinary diseases**	101913	32241	1.54 (1.51-1.57)	< 0.0001	139792	43731	1.51 (1.49-1.53)	< 0.0001	79467	27064	1.67 (1.64-1.70)	< 0.0001	68784	24331	1.71 (1.68-1.74)	< 0.0001
2	Bacterial infections	120550	12966	1.51 (1.47-1.56)	< 0.0001	166259	17575	1.54 (1.50-1.58)	< 0.0001	94345	11301	1.72 (1.67-1.76)	< 0.0001	82041	10357	1.78 (1.74-1.83)	< 0.0001
47	Respiratory tract infections	120869	10996	1.51 (1.47-1.56)	< 0.0001	166723	14971	1.62 (1.58-1.66)	< 0.0001	94584	10060	1.84 (1.78-1.90)	< 0.0001	82279	9276	1.92 (1.87-1.98)	< 0.0001
1	**Infectious diseases**	117400	20166	1.49 (1.46-1.53)	< 0.0001	162139	27339	1.50 (1.48-1.53)	< 0.0001	91744	17344	1.67 (1.63-1.70)	< 0.0001	79620	15850	1.73 (1.69-1.77)	< 0.0001
14	Anaemia	119031	15010	1.49 (1.45-1.52)	< 0.0001	164183	20154	1.47 (1.44-1.50)	< 0.0001	93127	12892	1.67 (1.63-1.71)	< 0.0001	80877	12026	1.77 (1.73-1.82)	< 0.0001
46	**Respiratory diseases**	112313	31280	1.48 (1.46-1.51)	< 0.0001	155312	42946	1.51 (1.49-1.53)	< 0.0001	87478	26706	1.67 (1.65-1.70)	< 0.0001	75753	24248	1.74 (1.71-1.77)	< 0.0001
43	*Cerebral infarction*	121342	3218	1.48 (1.40-1.56)	< 0.0001	167413	4428	1.45 (1.38-1.52)	< 0.0001	95048	2796	1.58 (1.50-1.67)	< 0.0001	82658	2632	1.68 (1.59-1.77)	< 0.0001
44	Arteriosclerosis	121812	1267	1.48 (1.36-1.62)	< 0.0001	167985	1742	1.60 (1.47-1.73)	< 0.0001	95433	1178	1.74 (1.59-1.90)	< 0.0001	83040	1155	1.97 (1.80-2.16)	< 0.0001
70	**Poisoning**	121084	1219	1.48 (1.36-1.61)	< 0.0001	167004	1649	1.54 (1.43-1.67)	< 0.0001	94822	1033	1.66 (1.52-1.81)	< 0.0001	82495	947	1.85 (1.70-2.03)	< 0.0001
13	**Blood diseases**	118569	16756	1.45 (1.42-1.48)	< 0.0001	163489	22563	1.44 (1.41-1.47)	< 0.0001	92728	14318	1.62 (1.58-1.66)	< 0.0001	80522	13227	1.70 (1.66-1.74)	< 0.0001
56	**Skin diseases**	113576	17798	1.44 (1.40-1.47)	< 0.0001	157162	24165	1.42 (1.40-1.45)	< 0.0001	88608	15315	1.61 (1.57-1.64)	< 0.0001	76947	13555	1.58 (1.54-1.61)	< 0.0001
17	**Mental and behavioural disorders**	118719	23311	1.43 (1.40-1.45)	< 0.0001	163912	32388	1.46 (1.44-1.49)	< 0.0001	92702	20016	1.60 (1.57-1.63)	< 0.0001	80535	18173	1.66 (1.63-1.69)	< 0.0001
22	Neurotic disorders	121215	7903	1.38 (1.33-1.42)	< 0.0001	167184	11212	1.44 (1.40-1.49)	< 0.0001	94920	6714	1.54 (1.49-1.60)	< 0.0001	82582	6031	1.57 (1.52-1.63)	< 0.0001
28	Transient ischaemic attack	121553	1385	1.35 (1.25-1.46)	< 0.0001	167639	1951	1.31 (1.22-1.40)	< 0.0001	95222	1165	1.39 (1.29-1.51)	< 0.0001	82861	1082	1.45 (1.33-1.57)	< 0.0001
50	**Digestive system diseases**	95804	47367	1.34 (1.32-1.36)	< 0.0001	132625	65568	1.33 (1.32-1.35)	< 0.0001	74273	38434	1.42 (1.40-1.44)	< 0.0001	63908	33948	1.45 (1.43-1.47)	< 0.0001
20	Psychotic disorders	121749	516	1.34 (1.18-1.52)	0.003	167893	746	1.64 (1.45-1.84)	< 0.0001	95355	500	1.81 (1.59-2.07)	< 0.0001	82984	451	1.88 (1.64-2.15)	< 0.0001
40	Cerebrovascular diseases	120769	6744	1.33 (1.28-1.38)	< 0.0001	166651	9494	1.37 (1.32-1.41)	< 0.0001	94582	5890	1.46 (1.41-1.51)	< 0.0001	82209	5560	1.55 (1.49-1.61)	< 0.0001

48	Chronic obstructive pulmonary disease	121080	7799	1.33 (1.28-1.37)	< 0.0001	167024	11130	1.53 (1.48-1.58)	< 0.0001	94724	7428	1.71 (1.65-1.77)	< 0.0001	82332	7013	1.83 (1.77-1.90)	< 0.0001
0	**All-cause mortality**	122001	13702	1.31 (1.28-1.35)	< 0.0001	168235	18817	1.37 (1.34-1.41)	< 0.0001	95604	12408	1.52 (1.48-1.56)	< 0.0001	83209	11690	1.61 (1.57-1.65)	< 0.0001
59	Rheumatoid arthritis	118415	18570	1.31 (1.28-1.34)	< 0.0001	163429	26491	1.31 (1.29-1.34)	< 0.0001	92603	15811	1.43 (1.40-1.46)	< 0.0001	80365	14486	1.49 (1.46-1.52)	< 0.0001
41	Stroke	121101	3955	1.31 (1.25-1.37)	< 0.0001	167066	5539	1.32 (1.27-1.38)	< 0.0001	94851	3423	1.40 (1.33-1.47)	< 0.0001	82485	3203	1.47 (1.40-1.55)	< 0.0001
19	Substance use disorders	120940	8876	1.30 (1.26-1.34)	< 0.0001	166813	12483	1.36 (1.33-1.40)	< 0.0001	94617	7816	1.45 (1.41-1.50)	< 0.0001	82284	7049	1.52 (1.48-1.57)	< 0.0001
73	**Self-harm**	121309	625	1.30 (1.16-1.46)	0.002	167316	851	1.40 (1.27-1.55)	< 0.0001	95018	535	1.48 (1.32-1.66)	< 0.0001	82678	482	1.67 (1.48-1.88)	< 0.0001
30	**Eye diseases**	116484	23717	1.24 (1.21-1.26)	< 0.0001	160495	34048	1.24 (1.22-1.26)	< 0.0001	90935	20100	1.32 (1.29-1.34)	< 0.0001	78881	18377	1.34 (1.32-1.37)	< 0.0001
26	Epilepsy	121440	1655	1.24 (1.15-1.33)	< 0.0001	167500	2294	1.32 (1.24-1.41)	< 0.0001	95140	1431	1.42 (1.32-1.52)	< 0.0001	82770	1340	1.53 (1.43-1.65)	< 0.0001
3	Viral infections	121161	1863	1.22 (1.14-1.30)	< 0.0001	167110	2518	1.18 (1.12-1.26)	< 0.0001	94921	1529	1.24 (1.16-1.33)	< 0.0001	82594	1366	1.28 (1.20-1.37)	< 0.0001
5	Colorectal cancer	121486	2309	1.21 (1.14-1.28)	< 0.0001	167471	3290	1.26 (1.20-1.33)	< 0.0001	95138	2010	1.34 (1.26-1.43)	< 0.0001	82794	1767	1.29 (1.21-1.37)	< 0.0001
31	**Ear diseases**	121334	945	1.21 (1.10-1.33)	0.01	167350	1309	1.24 (1.15-1.35)	< 0.0001	95076	778	1.28 (1.17-1.41)	< 0.0001	82734	701	1.33 (1.21-1.47)	< 0.0001
68	**Digestive and abdominal symptoms**	111863	22042	1.20 (1.18-1.23)	< 0.0001	154268	30880	1.23 (1.21-1.25)	< 0.0001	87383	18159	1.29 (1.27-1.32)	< 0.0001	75774	16290	1.33 (1.30-1.36)	< 0.0001
52	Inflammatory bowel disease	118489	5575	1.20 (1.15-1.24)	< 0.0001	163365	7832	1.24 (1.20-1.29)	< 0.0001	92656	4722	1.32 (1.27-1.38)	< 0.0001	80495	4286	1.38 (1.33-1.44)	< 0.0001
8	Breast cancer (Female)	62535	3171	1.15 (1.10-1.21)	< 0.0001	96269	4900	1.19 (1.14-1.24)	< 0.0001	49104	2543	1.19 (1.13-1.25)	< 0.0001	43217	2147	1.11 (1.05-1.17)	0.001
27	Headaches	121470	2004	1.15 (1.08-1.23)	0.002	167511	2762	1.14 (1.08-1.21)	0.0005	95169	1571	1.19 (1.12-1.27)	< 0.0001	82825	1353	1.14 (1.06-1.21)	0.050
72	**Fall**	118854	10306	1.12 (1.09-1.15)	< 0.0001	163839	15016	1.21 (1.18-1.24)	< 0.0001	92891	9045	1.28 (1.25-1.32)	< 0.0001	80794	8255	1.30 (1.26-1.34)	< 0.0001
51	Appendicitis	121439	698	1.12 (1.01-1.25)	1.00	167455	975	1.14 (1.04-1.24)	1.00	95168	540	1.12 (1.01-1.25)	1.00	82848	461	1.08 (0.97-1.21)	1.00
12	Leukaemia / Lymphoma	121601	3319	1.09 (1.04-1.14)	0.22	167671	4729	1.14 (1.09-1.19)	< 0.0001	95248	2863	1.20 (1.14-1.26)	< 0.0001	82901	2480	1.12 (1.07-1.18)	0.004
4	**Cancers**	116775	20966	1.07 (1.05-1.09)	< 0.0001	160526	29719	1.10 (1.08-1.12)	< 0.0001	91194	17641	1.15 (1.13-1.17)	< 0.0001	79309	15396	1.10 (1.08-1.13)	< 0.0001
69	**Injury**	115233	14186	1.06 (1.04-1.09)	< 0.0001	159238	20262	1.13 (1.11-1.15)	< 0.0001	90088	12055	1.18 (1.15-1.21)	< 0.0001	78396	10817	1.19 (1.16-1.21)	< 0.0001
18	Dementia	121976	2534	1.06 (1.00-1.12)	1.00	168199	3715	1.09 (1.04-1.14)	0.24	95579	2257	1.19 (1.12-1.26)	< 0.0001	83186	2257	1.31 (1.24-1.39)	< 0.0001
24	Parkinson disease	121946	1043	1.05 (0.96-1.14)	1.00	168154	1556	1.16 (1.08-1.26)	0.03	95561	948	1.21 (1.11-1.32)	0.009	83162	849	1.19 (1.09-1.30)	0.06
6	Lung cancer	121927	1690	0.99 (0.93-1.06)	1.00	168132	2593	1.12 (1.05-1.19)	0.07	95531	1564	1.14 (1.07-1.22)	0.04	83143	1456	1.19 (1.11-1.27)	0.0005
11	Brain cancer	121982	315	0.96 (0.82-1.11)	1.00	168208	436	0.98 (0.85-1.11)	1.00	95588	244	0.89 (0.76-1.05)	1.00	83193	206	0.79 (0.67-0.94)	1.00
42	*Intracerebral haemorrhage*	121747	980	0.94 (0.86-1.02)	1.00	167868	1456	1.04 (0.96-1.12)	1.00	95394	849	1.03 (0.94-1.12)	1.00	83023	775	1.05 (0.96-1.15)	1.00
9	Prostate cancer (Male)	57328	3135	0.92 (0.88-0.97)	0.02	68643	4145	0.96 (0.92-1.00)	0.64	44735	2601	0.93 (0.88-0.98)	0.07	38420	2285	0.90 (0.85-0.95)	0.001
7	Melanoma	120796	5370	0.85 (0.82-0.88)	< 0.0001	166415	7907	0.90 (0.87-0.93)	< 0.0001	94599	4493	0.90 (0.87-0.94)	< 0.0001	82329	3792	0.84 (0.81-0.87)	< 0.0001
25	Multiple sclerosis	121830	307	0.84 (0.73-0.97)	1.00	167960	484	1.00 (0.88-1.14)	1.00	95447	276	1.05 (0.90-1.22)	1.00	83067	223	1.00 (0.85-1.17)	1.00
71	**Road accidents**	121046	1279	0.74 (0.69-0.80)	< 0.0001	167020	1723	0.79 (0.75-0.84)	< 0.0001	94880	994	0.77 (0.72-0.84)	< 0.0001	82558	881	0.78 (0.72-0.85)	< 0.0001

Supplement eTable 4. Associations of BMI, WC, and WHtR with outcomes

		BMI				WC				WHtR			
		n	event	HR (95% CI)	P-value	n	event	HR (95% CI)	P-value	n	event	HR (95% CI)	P-value
29	Sleep disorders	493230	9956	1.93 (1.90-1.95)	< 0.0001	493230	9956	2.08 (2.05-2.11)	< 0.0001	493230	9956	2.09 (2.06-2.12)	< 0.0001
16	Diabetes mellitus	489001	39361	1.81 (1.80-1.82)	< 0.0001	489001	39361	2.01 (1.99-2.02)	< 0.0001	489001	39361	2.05 (2.03-2.06)	< 0.0001
60	Gout	494891	10362	1.62 (1.60-1.64)	< 0.0001	494891	10362	1.67 (1.64-1.70)	< 0.0001	494891	10362	1.68 (1.65-1.71)	< 0.0001
0.3	**Complex multimorbidity (4th disease)**	492109	76063	1.59 (1.58-1.60)	< 0.0001	492109	76063	1.66 (1.65-1.67)	< 0.0001	492109	76063	1.68 (1.67-1.69)	< 0.0001
0.2	**Third disease**	484794	115949	1.52 (1.51-1.53)	< 0.0001	484794	115949	1.57 (1.56-1.58)	< 0.0001	484794	115949	1.58 (1.57-1.59)	< 0.0001
15	**Endocrine diseases**	467853	142086	1.52 (1.51-1.53)	< 0.0001	467853	142086	1.55 (1.55-1.56)	< 0.0001	467853	142086	1.57 (1.57-1.58)	< 0.0001
39	Heart failure	493856	19152	1.49 (1.47-1.51)	< 0.0001	493856	19152	1.55 (1.53-1.57)	< 0.0001	493856	19152	1.56 (1.54-1.58)	< 0.0001
33	Hypertension	467784	132767	1.48 (1.48-1.49)	< 0.0001	467784	132767	1.51 (1.51-1.52)	< 0.0001	467784	132767	1.55 (1.54-1.55)	< 0.0001
0.1	**Simple multimorbidity (2nd disease)**	463681	166774	1.43 (1.42-1.44)	< 0.0001	463681	166774	1.46 (1.45-1.46)	< 0.0001	463681	166774	1.47 (1.46-1.48)	< 0.0001
66	Renal failure	494325	41055	1.42 (1.41-1.43)	< 0.0001	494325	41055	1.47 (1.45-1.48)	< 0.0001	494325	41055	1.48 (1.47-1.49)	< 0.0001
53	Liver diseases	494013	15857	1.42 (1.40-1.44)	< 0.0001	494013	15857	1.51 (1.49-1.53)	< 0.0001	494013	15857	1.53 (1.51-1.55)	< 0.0001
61	Osteoarthritis	478245	82728	1.41 (1.40-1.42)	< 0.0001	478245	82728	1.39 (1.38-1.40)	< 0.0001	478245	82728	1.39 (1.38-1.40)	< 0.0001
57	Eczema and skin infections	487834	23693	1.41 (1.39-1.42)	< 0.0001	487834	23693	1.45 (1.44-1.47)	< 0.0001	487834	23693	1.44 (1.43-1.46)	< 0.0001
37	Pulmonary embolism	494283	8967	1.37 (1.34-1.39)	< 0.0001	494283	8967	1.41 (1.38-1.44)	< 0.0001	494283	8967	1.36 (1.33-1.39)	< 0.0001
55	Pancreatitis	494758	3112	1.37 (1.33-1.41)	< 0.0001	494758	3112	1.43 (1.38-1.47)	< 0.0001	494758	3112	1.44 (1.39-1.48)	< 0.0001
32	**Circulatory diseases**	435012	175304	1.35 (1.34-1.36)	< 0.0001	435012	175304	1.37 (1.36-1.37)	< 0.0001	435012	175304	1.38 (1.37-1.38)	< 0.0001
35	*Angina pectoris*	486123	23932	1.33 (1.32-1.35)	< 0.0001	486123	23932	1.35 (1.33-1.36)	< 0.0001	486123	23932	1.40 (1.38-1.42)	< 0.0001
34	Ischaemic heart disease	481099	48187	1.31 (1.30-1.32)	< 0.0001	481099	48187	1.33 (1.32-1.34)	< 0.0001	481099	48187	1.36 (1.35-1.37)	< 0.0001
38	Arrhythmias	489334	43957	1.30 (1.29-1.31)	< 0.0001	489334	43957	1.34 (1.33-1.35)	< 0.0001	489334	43957	1.28 (1.26-1.29)	< 0.0001
45	Deep vein thrombosis	493390	7844	1.29 (1.26-1.31)	< 0.0001	493390	7844	1.32 (1.30-1.35)	< 0.0001	493390	7844	1.28 (1.25-1.31)	< 0.0001
23	**Neurological disorders**	474833	66201	1.28 (1.27-1.29)	< 0.0001	474833	66201	1.29 (1.28-1.30)	< 0.0001	474833	66201	1.30 (1.29-1.31)	< 0.0001
10	Kidney cancer	495495	2236	1.28 (1.23-1.33)	< 0.0001	495495	2236	1.31 (1.26-1.37)	< 0.0001	495495	2236	1.29 (1.24-1.34)	< 0.0001
21	Mood disorders	491472	29698	1.27 (1.26-1.29)	< 0.0001	491472	29698	1.33 (1.31-1.34)	< 0.0001	491472	29698	1.34 (1.32-1.35)	< 0.0001
49	Asthma	483992	37341	1.27 (1.26-1.28)	< 0.0001	483992	37341	1.32 (1.31-1.33)	< 0.0001	483992	37341	1.35 (1.34-1.36)	< 0.0001
63	Backpain	487592	26519	1.26 (1.25-1.27)	< 0.0001	487592	26519	1.29 (1.27-1.30)	< 0.0001	487592	26519	1.29 (1.28-1.31)	< 0.0001
58	**Musculoskeletal diseases**	438286	150109	1.25 (1.25-1.26)	< 0.0001	438286	150109	1.25 (1.24-1.26)	< 0.0001	438286	150109	1.25 (1.25-1.26)	< 0.0001
62	Sciatica	491506	15123	1.25 (1.24-1.27)	< 0.0001	491506	15123	1.25 (1.23-1.27)	< 0.0001	491506	15123	1.25 (1.23-1.27)	< 0.0001
54	Alcohol-related liver diseases	495531	1701	1.25 (1.20-1.30)	< 0.0001	495531	1701	1.42 (1.37-1.49)	< 0.0001	495531	1701	1.50 (1.44-1.56)	< 0.0001
64	Soft tissue diseases	475676	48579	1.24 (1.23-1.25)	< 0.0001	475676	48579	1.24 (1.23-1.25)	< 0.0001	475676	48579	1.25 (1.24-1.26)	< 0.0001
36	*Myocardial infarction*	490725	13883	1.23 (1.21-1.25)	< 0.0001	490725	13883	1.24 (1.22-1.26)	< 0.0001	490725	13883	1.28 (1.26-1.30)	< 0.0001
47	Respiratory tract infections	492365	32236	1.23 (1.21-1.24)	< 0.0001	492365	32236	1.30 (1.28-1.31)	< 0.0001	492365	32236	1.31 (1.30-1.32)	< 0.0001
2	Bacterial infections	491205	39490	1.21 (1.20-1.22)	< 0.0001	491205	39490	1.26 (1.25-1.27)	< 0.0001	491205	39490	1.27 (1.26-1.28)	< 0.0001
67	**Circulatory and respiratory symptoms**	465131	68898	1.21 (1.20-1.21)	< 0.0001	465131	68898	1.23 (1.22-1.24)	< 0.0001	465131	68898	1.24 (1.23-1.25)	< 0.0001
1	**Infectious diseases**	481279	62795	1.20 (1.20-1.21)	< 0.0001	481279	62795	1.24 (1.23-1.25)	< 0.0001	481279	62795	1.25 (1.24-1.26)	< 0.0001
56	**Skin diseases**	469380	58065	1.20 (1.20-1.21)	< 0.0001	469380	58065	1.23 (1.22-1.24)	< 0.0001	469380	58065	1.22 (1.21-1.23)	< 0.0001
65	**Genitourinary diseases**	424122	105547	1.20 (1.20-1.21)	< 0.0001	424122	105547	1.23 (1.22-1.24)	< 0.0001	424122	105547	1.23 (1.23-1.24)	< 0.0001
14	Anaemia	485916	45783	1.20 (1.19-1.21)	< 0.0001	485916	45783	1.23 (1.22-1.24)	< 0.0001	485916	45783	1.25 (1.24-1.26)	< 0.0001
44	Arteriosclerosis	495381	3744	1.20 (1.16-1.24)	< 0.0001	495381	3744	1.28 (1.24-1.32)	< 0.0001	495381	3744	1.32 (1.28-1.36)	< 0.0001
46	**Respiratory diseases**	465724	101286	1.19 (1.18-1.20)	< 0.0001	465724	101286	1.24 (1.23-1.25)	< 0.0001	465724	101286	1.25 (1.25-1.26)	< 0.0001
70	**Poisoning**	493033	3722	1.19 (1.15-1.22)	< 0.0001	493033	3722	1.24 (1.20-1.27)	< 0.0001	493033	3722	1.27 (1.23-1.31)	< 0.0001
13	**Blood diseases**	484225	52131	1.18 (1.17-1.19)	< 0.0001	484225	52131	1.22 (1.21-1.23)	< 0.0001	484225	52131	1.23 (1.22-1.24)	< 0.0001
43	*Cerebral infarction*	494133	10211	1.18 (1.16-1.21)	< 0.0001	494133	10211	1.21 (1.18-1.23)	< 0.0001	494133	10211	1.23 (1.20-1.25)	< 0.0001
17	**Mental and behavioural disorders**	486370	76820	1.17 (1.16-1.18)	< 0.0001	486370	76820	1.21 (1.20-1.22)	< 0.0001	486370	76820	1.23 (1.22-1.24)	< 0.0001
0	**All-cause mortality**	495900	42753	1.16 (1.15-1.18)	< 0.0001	495900	42753	1.22 (1.21-1.23)	< 0.0001	495900	42753	1.23 (1.22-1.24)	< 0.0001
48	Chronic obstructive pulmonary disease	493661	23358	1.16 (1.15-1.18)	< 0.0001	493661	23358	1.27 (1.25-1.28)	< 0.0001	493661	23358	1.30 (1.29-1.32)	< 0.0001
22	Neurotic disorders	493541	26384	1.15 (1.14-1.16)	< 0.0001	493541	26384	1.20 (1.18-1.21)	< 0.0001	493541	26384	1.20 (1.19-1.22)	< 0.0001
59	Rheumatoid arthritis	485066	62314	1.15 (1.14-1.16)	< 0.0001	485066	62314	1.17 (1.16-1.18)	< 0.0001	485066	62314	1.19 (1.18-1.19)	< 0.0001
20	Psychotic disorders	495210	1585	1.15 (1.10-1.20)	< 0.0001	495210	1585	1.26 (1.21-1.32)	< 0.0001	495210	1585	1.28 (1.23-1.34)	< 0.0001
40	Cerebrovascular diseases	492411	22282	1.14 (1.13-1.15)	< 0.0001	492411	22282	1.17 (1.16-1.19)	< 0.0001	492411	22282	1.20 (1.18-1.21)	< 0.0001
41	Stroke	493269	13313	1.13 (1.12-1.15)	< 0.0001	493269	13313	1.16 (1.14-1.18)	< 0.0001	493269	13313	1.17 (1.15-1.19)	< 0.0001
50	**Digestive system diseases**	406095	174068	1.13 (1.12-1.13)	< 0.0001	406095	174068	1.15 (1.15-1.16)	< 0.0001	406095	174068	1.16 (1.15-1.17)	< 0.0001
19	Substance use disorders	492516	30983	1.12 (1.11-1.13)	< 0.0001	492516	30983	1.16 (1.15-1.17)	< 0.0001	492516	30983	1.18 (1.17-1.20)	< 0.0001
28	Transient ischaemic attack	494579	4802	1.12 (1.09-1.15)	< 0.0001	494579	4802	1.14 (1.11-1.18)	< 0.0001	494579	4802	1.16 (1.12-1.19)	< 0.0001
73	**Self-harm**	493783	2087	1.12 (1.08-1.17)	< 0.0001	493783	2087	1.18 (1.13-1.23)	< 0.0001	493783	2087	1.22 (1.17-1.27)	< 0.0001
26	Epilepsy	494157	5544	1.11 (1.08-1.13)	< 0.0001	494157	5544	1.14 (1.11-1.17)	< 0.0001	494157	5544	1.17 (1.14-1.20)	< 0.0001
30	**Eye diseases**	476556	84729	1.10 (1.09-1.11)	< 0.0001	476556	84729	1.12 (1.12-1.13)	< 0.0001	476556	84729	1.13 (1.13-1.14)	< 0.0001
52	Inflammatory bowel disease	484041	19742	1.09 (1.08-1.11)	< 0.0001	484041	19742	1.13 (1.11-1.14)	< 0.0001	484041	19742	1.14 (1.13-1.16)	< 0.0001
5	Colorectal cancer	493983	8227	1.09 (1.07-1.11)	< 0.0001	493983	8227	1.13 (1.10-1.15)	< 0.0001	493983	8227	1.11 (1.08-1.13)	< 0.0001
68	**Digestive and abdominal symptoms**	460697	79339	1.08 (1.08-1.09)	< 0.0001	460697	79339	1.11 (1.10-1.12)	< 0.0001	460697	79339	1.12 (1.11-1.13)	< 0.0001
3	Viral infections	492884	6574	1.08 (1.06-1.11)	< 0.0001	492884	6574	1.10 (1.07-1.12)	< 0.0001	492884	6574	1.11 (1.08-1.13)	< 0.0001
31	**Ear diseases**	493777	3410	1.08 (1.04-1.11)	0.0003	493777	3410	1.10 (1.07-1.14)	< 0.0001	493777	3410	1.11 (1.08-1.15)	< 0.0001
72	**Fall**	485173	36667	1.07 (1.06-1.09)	< 0.0001	485173	36667	1.12 (1.11-1.14)	< 0.0001	485173	36667	1.12 (1.11-1.13)	< 0.0001
27	Headaches	494187	7391	1.06 (1.04-1.09)	< 0.0001	494187	7391	1.07 (1.05-1.10)	< 0.0001	494187	7391	1.06 (1.04-1.09)	< 0.0001
8	Breast cancer (Female)	262868	12224	1.06 (1.04-1.08)	< 0.0001	262868	12224	1.08 (1.06-1.10)	< 0.0001	262868	12224	1.05 (1.03-1.07)	< 0.0001
18	Dementia	495824	9244	1.05 (1.03-1.07)	0.0005	495824	9244	1.08 (1.05-1.10)	< 0.0001	495824	9244	1.12 (1.10-1.15)	< 0.0001
51	Appendicitis	493676	2753	1.05 (1.01-1.09)	0.09	493676	2753	1.06 (1.02-1.10)	0.04	493676	2753	1.04 (1.00-1.08)	0.44
69	**Injury**	471296	53225	1.04 (1.03-1.05)	< 0.0001	471296	53225	1.08 (1.07-1.09)	< 0.0001	471296	53225	1.07 (1.06-1.08)	< 0.0001
12	Leukaemia / Lymphoma	494411	12409	1.04 (1.02-1.06)	0.0001	494411	12409	1.08 (1.06-1.10)	< 0.0001	494411	12409	1.05 (1.03-1.07)	< 0.0001
4	**Cancers**	475215	80417	1.03 (1.03-1.04)	< 0.0001	475215	80417	1.06 (1.05-1.06)	< 0.0001	475215	80417	1.03 (1.03-1.04)	< 0.0001
24	Parkinson disease	495694	4058	1.02 (0.98-1.05)	1.00	495694	4058	1.07 (1.03-1.10)	0.002	495694	4058	1.06 (1.03-1.09)	0.005
42	*Intracerebral haemorrhage*	494971	3905	1.01 (0.97-1.04)	1.00	494971	3905	1.03 (1.00-1.07)	0.46	494971	3905	1.04 (1.00-1.07)	0.30
6	Lung cancer	495622	6298	0.98 (0.95-1.00)	0.64	495622	6298	1.05 (1.03-1.08)	0.002	495622	6298	1.05 (1.03-1.08)	0.002
11	Brain cancer	495805	1287	0.98 (0.93-1.04)	1.00	495805	1287	0.98 (0.93-1.04)	1.00	495805	1287	0.97 (0.91-1.02)	1.00
9	Prostate cancer (Male)	224487	13355	0.96 (0.94-0.98)	< 0.0001	224487	13355	0.98 (0.96-0.99)	0.010	224487	13355	0.96 (0.95-0.98)	< 0.0001
7	Melanoma	490441	24183	0.94 (0.92-0.95)	< 0.0001	490441	24183	0.95 (0.94-0.97)	< 0.0001	490441	24183	0.92 (0.91-0.94)	< 0.0001
25	Multiple sclerosis	495244	1341	0.93 (0.88-0.98)	0.12	495244	1341	1.02 (0.96-1.07)	1.00	495244	1341	1.00 (0.95-1.06)	1.00
71	**Road accidents**	491968	5978	0.90 (0.87-0.92)	< 0.0001	491968	5978	0.90 (0.88-0.92)	< 0.0001	491968	5978	0.87 (0.84-0.89)	< 0.0001

Supplement eTable 5. Difference in population attributable fractions between obesity defined using different measures

		PAF_{WC} - PAF_{BMI}			PAF_{WHtR} - PAF_{BMI}		
		**%**	**Lower CI**	**Upper CI**	**%**	**Lower CI**	**Upper CI**
0	**All-cause mortality**	0.18	-1.97	2.34	0.19	-1.97	2.35
0.1	**Simple multimorbidity (2nd disease)**	0.06	-0.76	0.87	0.07	-0.75	0.88
0.2	**Third disease**	0.14	-0.85	1.13	0.15	-0.84	1.14
0.3	**Complex multimorbidity (4th disease)**	0.17	-1.00	1.35	0.19	-0.99	1.37
1	**Infectious diseases**	0.14	-1.46	1.74	0.15	-1.45	1.75
2	Bacterial infections	0.11	-1.95	2.17	0.12	-1.94	2.18
3	Viral infections	0.59	-4.65	5.83	0.58	-4.66	5.82
4	**Cancers**	0.10	-1.39	1.59	0.10	-1.39	1.59
5	Colorectal cancer	0.15	-4.99	5.29	0.14	-5.00	5.28
6	Lung cancer	0.04	-6.05	6.12	0.03	-6.05	6.12
7	Melanoma	-0.01	-2.94	2.93	-0.01	-2.94	2.92
8	Breast cancer (Female)	0.06	-3.06	3.19	0.06	-3.06	3.19
9	Prostate cancer (Male)	-0.04	-5.35	5.27	-0.11	-5.42	5.20
10	Kidney cancer	-0.31	-9.37	8.74	-0.32	-9.37	8.74
11	Brain cancer	-0.11	-14.53	14.31	-0.11	-14.53	14.31
12	Leukaemia / Lymphoma	0.20	-3.95	4.36	0.20	-3.96	4.36
13	**Blood diseases**	0.03	-1.73	1.79	0.05	-1.72	1.81
14	Anaemia	0.03	-1.85	1.91	0.05	-1.83	1.93
15	**Endocrine diseases**	0.08	-0.78	0.95	0.09	-0.77	0.96
16	Diabetes mellitus	0.19	-0.90	1.28	0.19	-0.90	1.28
17	**Mental and behavioural disorders**	0.06	-1.32	1.43	0.07	-1.31	1.44
18	Dementia	0.08	-5.02	5.17	0.07	-5.02	5.17
19	Substance use disorders	0.11	-2.03	2.26	0.11	-2.03	2.25
20	Psychotic disorders	0.52	-9.67	10.70	0.51	-9.67	10.70
21	Mood disorders	0.16	-1.99	2.32	0.18	-1.98	2.33
22	Neurotic disorders	0.17	-2.24	2.59	0.17	-2.25	2.59
23	**Neurological disorders**	0.05	-1.41	1.52	0.08	-1.38	1.55
24	Parkinson disease	0.00	-8.02	8.02	0.00	-8.02	8.02
25	Multiple sclerosis	0.25	-10.16	10.66	0.25	-10.16	10.66
26	Epilepsy	-0.17	-6.03	5.70	-0.17	-6.04	5.69
27	Headaches	0.41	-4.14	4.96	0.40	-4.15	4.95
28	Transient ischaemic attack	-0.27	-6.93	6.40	-0.27	-6.93	6.40
29	Sleep disorders	-0.03	-2.23	2.17	0.00	-2.20	2.20
30	**Eye diseases**	0.01	-1.38	1.40	0.02	-1.37	1.41
31	**Ear diseases**	0.37	-7.02	7.77	0.52	-6.87	7.91
32	**Circulatory diseases**	0.03	-0.75	0.82	0.03	-0.75	0.82
33	Hypertension	0.03	-0.89	0.96	0.04	-0.89	0.96
34	Ischaemic heart disease	0.18	-1.65	2.02	0.18	-1.65	2.02
35	*Angina pectoris*	0.21	-2.36	2.78	0.21	-2.37	2.78
36	*Myocardial infarction*	0.12	-3.67	3.91	0.11	-3.68	3.91
37	Pulmonary embolism	0.19	-3.93	4.31	0.19	-3.93	4.30
38	Arrhythmias	0.07	-1.90	2.04	0.05	-1.92	2.03
39	Heart failure	-0.08	-2.75	2.58	-0.09	-2.75	2.58
40	Cerebrovascular diseases	0.07	-3.01	3.15	0.06	-3.02	3.14
41	Stroke	0.24	-3.80	4.28	0.24	-3.80	4.28
42	*Intracerebral haemorrhage*	0.41	-7.23	8.05	0.41	-7.24	8.05
43	*Cerebral infarction*	0.20	-4.37	4.77	0.20	-4.38	4.77
44	Arteriosclerosis	0.48	-7.14	8.10	0.48	-7.15	8.10
45	Deep vein thrombosis	0.24	-4.34	4.82	0.24	-4.34	4.82
46	**Respiratory diseases**	0.16	-1.04	1.36	0.17	-1.03	1.36
47	Respiratory tract infections	0.14	-2.23	2.52	0.19	-2.18	2.56
48	Chronic obstructive pulmonary disease	0.44	-2.36	3.23	0.44	-2.36	3.23
49	Asthma	0.24	-1.66	2.15	0.24	-1.66	2.15
50	**Digestive system diseases**	0.06	-0.73	0.86	0.07	-0.72	0.86
51	Appendicitis	-0.03	-7.58	7.53	-0.03	-7.59	7.52
52	Inflammatory bowel disease	0.28	-2.68	3.24	0.30	-2.66	3.26

53	Liver diseases	0.21	-2.57	2.99	0.24	-2.54	3.01
54	Alcohol-related liver diseases	0.60	-8.86	10.07	0.60	-8.87	10.07
55	Pancreatitis	-0.36	-7.00	6.28	-0.36	-7.00	6.28
56	**Skin diseases**	0.19	-1.43	1.82	0.20	-1.43	1.83
57	Eczema and skin infections	0.38	-2.00	2.76	0.37	-2.01	2.76
58	**Musculoskeletal diseases**	0.01	-0.85	0.87	0.02	-0.85	0.88
59	Rheumatoid arthritis	0.21	-1.33	1.74	0.22	-1.32	1.76
60	Gout	-0.06	-2.95	2.83	-0.06	-2.95	2.83
61	Osteoarthritis	-0.02	-1.23	1.19	-0.01	-1.22	1.21
62	Sciatica	-0.05	-3.23	3.13	0.01	-3.17	3.19
63	Backpain	0.03	-2.35	2.41	0.08	-2.30	2.46
64	Soft tissue diseases	0.01	-1.71	1.73	0.03	-1.70	1.75
65	**Genitourinary diseases**	-0.01	-1.16	1.13	-0.01	-1.16	1.13
66	Renal failure	0.10	-1.75	1.96	0.12	-1.73	1.98
67	**Circulatory and respiratory symptoms**	0.07	-1.40	1.54	0.07	-1.40	1.54
68	**Digestive and abdominal symptoms**	0.03	-1.32	1.39	0.05	-1.31	1.40
69	**Injury**	0.15	-1.62	1.91	0.17	-1.60	1.94
70	**Poisoning**	0.69	-5.86	7.25	0.69	-5.86	7.25
71	**Road accidents**	0.24	-4.99	5.48	0.24	-5.00	5.48
72	**Fall**	0.15	-2.02	2.31	0.17	-2.00	2.34
73	**Self-harm**	0.19	-8.34	8.72	0.19	-8.34	8.72

Supplement eTable 6. Difference in population attributable fractions between obesity class defined using different measures

		BMI 30-34 and equivalents						BMI ≥35 and equivalents					
		PAF_{WC} - PAF_{BMI}			PAF_{WHtR} - PAF_{BMI}			PAF_{WC} - PAF_{BMI}			PAF_{WHtR} - PAF_{BMI}		
		%	Lower CI	Upper CI	%	Lower CI	Upper CI	%	Lower CI	Upper CI	%	Lower CI	Upper CI
0	**All-cause mortality**	0.25	-1.63	2.14	0.27	-1.62	2.15	0.00	-1.57	1.56	0.00	-1.57	1.56
0.1	**Simple multimorbidity (2nd disease)**	0.10	-0.71	0.91	0.11	-0.70	0.92	0.00	-0.75	0.76	0.00	-0.75	0.76
0.2	**Third disease**	0.26	-0.86	1.38	0.27	-0.85	1.39	0.00	-1.05	1.05	0.00	-1.05	1.05
0.3	**Complex multimorbidity (4th disease)**	0.36	-1.13	1.84	0.37	-1.12	1.85	0.00	-1.33	1.33	0.00	-1.33	1.33
1	**Infectious diseases**	0.25	-1.17	1.67	0.27	-1.15	1.69	0.00	-1.21	1.21	0.00	-1.21	1.21
2	Bacterial infections	0.30	-1.57	2.16	0.33	-1.54	2.19	0.00	-1.60	1.60	0.00	-1.60	1.60
3	Viral infections	0.38	-3.86	4.62	0.38	-3.86	4.63	0.00	-3.63	3.63	0.00	-3.63	3.63
4	**Cancers**	0.07	-1.09	1.23	0.06	-1.10	1.22	0.00	-0.91	0.91	0.00	-0.91	0.91
5	Colorectal cancer	-0.01	-4.23	4.20	-0.01	-4.22	4.21	0.00	-3.43	3.43	0.00	-3.43	3.43
6	Lung cancer	-0.04	-4.79	4.71	-0.04	-4.78	4.71	0.00	-3.80	3.80	0.00	-3.80	3.80
7	Melanoma	0.00	-2.12	2.11	-0.03	-2.14	2.09	0.00	-1.52	1.52	0.00	-1.52	1.52
8	Breast cancer (Female)	0.15	-2.26	2.55	0.13	-2.28	2.53	0.00	-2.13	2.13	0.00	-2.13	2.13
9	Prostate cancer (Male)	0.05	-3.82	3.91	-0.02	-3.88	3.85	0.00	-2.63	2.63	0.00	-2.63	2.63
10	Kidney cancer	-0.43	-9.56	8.69	-0.43	-9.56	8.69	0.00	-7.80	7.80	0.00	-7.80	7.80
11	Brain cancer	-0.21	-10.72	10.30	-0.21	-10.72	10.30	0.00	-7.65	7.65	0.00	-7.65	7.65
12	Leukaemia / Lymphoma	0.17	-3.11	3.45	0.13	-3.15	3.41	0.00	-2.60	2.59	0.00	-2.60	2.59
13	**Blood diseases**	0.12	-1.44	1.67	0.13	-1.43	1.69	0.00	-1.32	1.32	0.00	-1.32	1.32
14	Anaemia	0.10	-1.58	1.79	0.12	-1.57	1.81	0.00	-1.44	1.44	0.00	-1.44	1.44
15	**Endocrine diseases**	0.13	-0.80	1.06	0.14	-0.79	1.08	0.00	-0.89	0.89	0.00	-0.89	0.89
16	Diabetes mellitus	0.38	-1.50	2.26	0.38	-1.50	2.26	0.00	-1.50	1.50	0.00	-1.50	1.50
17	**Mental and behavioural disorders**	0.06	-1.13	1.25	0.07	-1.12	1.26	0.00	-1.02	1.02	0.00	-1.02	1.02
18	Dementia	0.20	-3.92	4.32	0.21	-3.91	4.33	0.00	-3.30	3.30	0.00	-3.30	3.30
19	Substance use disorders	0.04	-1.73	1.81	0.04	-1.73	1.81	0.00	-1.48	1.48	0.00	-1.48	1.48
20	Psychotic disorders	0.78	-8.18	9.75	0.78	-8.18	9.75	0.00	-7.87	7.87	0.00	-7.87	7.87
21	Mood disorders	0.14	-1.92	2.21	0.16	-1.90	2.23	0.00	-1.83	1.83	0.00	-1.83	1.83
22	Neurotic disorders	0.24	-1.82	2.31	0.25	-1.82	2.31	0.00	-1.81	1.81	0.00	-1.81	1.81
23	**Neurological disorders**	0.03	-1.32	1.38	0.08	-1.27	1.43	0.00	-1.17	1.17	0.00	-1.17	1.17
24	Parkinson disease	-0.17	-6.50	6.17	-0.16	-6.50	6.17	0.00	-4.91	4.91	0.00	-4.91	4.91
25	Multiple sclerosis	0.40	-6.93	7.72	0.40	-6.92	7.72	0.00	-6.00	5.99	0.00	-6.00	5.99
26	Epilepsy	0.09	-4.85	5.03	0.09	-4.85	5.03	0.00	-4.15	4.15	0.00	-4.15	4.15
27	Headaches	0.34	-3.14	3.83	0.35	-3.13	3.83	0.00	-3.08	3.08	0.00	-3.08	3.08
28	Transient ischaemic attack	-0.56	-6.30	5.18	-0.56	-6.29	5.18	0.00	-4.83	4.82	0.00	-4.83	4.82
29	Sleep disorders	-0.15	-4.27	3.98	-0.07	-4.20	4.05	0.00	-2.51	2.51	0.00	-2.51	2.51
30	**Eye diseases**	-0.01	-1.16	1.13	0.01	-1.14	1.15	0.01	-0.94	0.96	0.01	-0.94	0.96
31	**Ear diseases**	0.70	-5.34	6.74	0.85	-5.18	6.89	0.00	-4.92	4.92	0.00	-4.92	4.92
32	**Circulatory diseases**	0.07	-0.65	0.80	0.08	-0.65	0.80	0.00	-0.65	0.65	0.00	-0.65	0.65
33	Hypertension	0.12	-0.88	1.11	0.13	-0.86	1.13	0.00	-0.95	0.95	0.00	-0.95	0.95
34	Ischaemic heart disease	0.36	-1.48	2.19	0.36	-1.48	2.19	0.00	-1.63	1.63	0.00	-1.63	1.63
35	*Angina pectoris*	0.36	-2.37	3.09	0.36	-2.37	3.09	0.00	-2.49	2.48	0.00	-2.49	2.48
36	*Myocardial infarction*	0.34	-3.24	3.92	0.34	-3.24	3.92	0.00	-3.17	3.17	0.00	-3.17	3.17
37	Pulmonary embolism	0.18	-4.24	4.61	0.11	-4.31	4.54	0.00	-3.81	3.81	0.00	-3.81	3.81
38	Arrhythmias	0.04	-1.82	1.90	0.00	-1.86	1.86	0.00	-1.57	1.57	0.00	-1.57	1.57
39	Heart failure	-0.19	-3.36	2.98	-0.19	-3.36	2.99	0.00	-2.55	2.55	0.00	-2.55	2.55
40	Cerebrovascular diseases	0.10	-2.58	2.77	0.10	-2.57	2.77	0.00	-2.24	2.24	0.00	-2.24	2.24
41	Stroke	0.23	-3.21	3.66	0.23	-3.21	3.66	0.00	-2.83	2.83	0.00	-2.83	2.83
42	*Intracerebral haemorrhage*	0.33	-5.40	6.07	0.33	-5.40	6.07	0.00	-4.57	4.57	0.00	-4.57	4.57
43	*Cerebral infarction*	0.13	-3.98	4.24	0.13	-3.98	4.24	0.00	-3.42	3.42	0.00	-3.42	3.42
44	Arteriosclerosis	0.41	-6.73	7.54	0.41	-6.73	7.54	0.00	-6.07	6.07	0.00	-6.07	6.07
45	Deep vein thrombosis	0.61	-3.86	5.09	0.62	-3.86	5.09	0.00	-3.90	3.90	0.00	-3.90	3.90
46	**Respiratory diseases**	0.21	-0.84	1.27	0.22	-0.84	1.27	0.00	-0.89	0.89	0.00	-0.89	0.89
47	Respiratory tract infections	0.24	-2.00	2.49	0.30	-1.94	2.54	0.00	-1.90	1.90	0.00	-1.90	1.90
48	Chronic obstructive pulmonary disease	0.76	-1.78	3.31	0.79	-1.76	3.33	0.00	-2.18	2.18	0.00	-2.18	2.18
49	Asthma	0.30	-1.53	2.12	0.30	-1.52	2.13	0.01	-1.58	1.61	0.01	-1.58	1.61
50	**Digestive system diseases**	0.11	-0.54	0.75	0.11	-0.53	0.75	0.00	-0.51	0.52	0.00	-0.51	0.52
51	Appendicitis	-0.11	-5.97	5.74	-0.11	-5.96	5.74	0.00	-4.63	4.63	0.00	-4.63	4.63
52	Inflammatory bowel disease	0.33	-2.09	2.76	0.36	-2.06	2.79	0.00	-2.04	2.04	0.00	-2.04	2.04
53	Liver diseases	0.40	-2.87	3.67	0.40	-2.87	3.67	0.00	-2.84	2.84	0.00	-2.84	2.84
54	Alcohol-related liver diseases	1.06	-8.77	10.89	1.06	-8.77	10.89	0.00	-8.80	8.80	0.00	-8.80	8.80
55	Pancreatitis	-0.38	-7.67	6.92	-0.38	-7.67	6.92	0.00	-6.58	6.58	0.00	-6.58	6.58
56	**Skin diseases**	0.18	-1.23	1.60	0.18	-1.23	1.60	0.00	-1.19	1.19	0.00	-1.19	1.19
57	Eczema and skin infections	0.40	-2.09	2.90	0.38	-2.12	2.88	0.00	-2.08	2.08	0.00	-2.08	2.08
58	**Musculoskeletal diseases**	0.01	-0.75	0.76	0.02	-0.73	0.78	0.00	-0.64	0.64	0.00	-0.64	0.64
59	Rheumatoid arthritis	0.27	-1.02	1.57	0.30	-1.00	1.59	0.01	-1.11	1.12	0.01	-1.11	1.12
60	Gout	-0.23	-4.43	3.97	-0.23	-4.44	3.97	0.00	-3.72	3.72	0.00	-3.72	3.72
61	Osteoarthritis	0.00	-1.23	1.23	0.02	-1.21	1.25	0.01	-1.11	1.13	0.01	-1.11	1.13
62	Sciatica	-0.21	-3.17	2.75	-0.14	-3.10	2.82	0.00	-2.62	2.62	0.00	-2.62	2.62
63	Backpain	0.14	-2.10	2.38	0.16	-2.08	2.40	0.00	-1.98	1.98	0.00	-1.98	1.98
64	Soft tissue diseases	0.07	-1.50	1.64	0.10	-1.48	1.67	0.00	-1.34	1.34	0.00	-1.34	1.34
65	**Genitourinary diseases**	-0.01	-1.00	0.98	0.00	-0.99	0.99	0.00	-0.84	0.83	0.00	-0.84	0.83
66	Renal failure	0.24	-1.84	2.31	0.27	-1.81	2.35	0.00	-1.81	1.81	0.00	-1.81	1.81
67	**Circulatory and respiratory symptoms**	0.08	-1.23	1.38	0.09	-1.22	1.39	0.00	-1.12	1.12	0.00	-1.12	1.12
68	**Digestive and abdominal symptoms**	0.10	-0.99	1.19	0.11	-0.98	1.20	0.00	-0.90	0.89	0.00	-0.90	0.89
69	**Injury**	0.15	-1.23	1.53	0.16	-1.21	1.54	0.00	-1.11	1.11	0.00	-1.11	1.11
70	**Poisoning**	0.93	-4.83	6.69	0.93	-4.83	6.69	0.00	-5.00	5.00	0.00	-5.00	5.00
71	**Road accidents**	0.25	-3.28	3.79	0.18	-3.35	3.71	0.00	-2.55	2.56	0.00	-2.55	2.56
72	**Fall**	0.15	-1.59	1.90	0.18	-1.56	1.93	0.00	-1.45	1.45	0.00	-1.45	1.45
73	**Self-harm**	0.12	-6.88	7.11	0.12	-6.87	7.11	0.00	-6.00	6.00	0.00	-6.00	6.00

Supplement eTable 7. Difference in population attributable fractions between overweight defined using different measures

		PAF_{WC} - PAF_{BMI}			PAF_{WHtR} - PAF_{BMI}		
		%	Lower CI	Upper CI	%	Lower CI	Upper CI
0	**All-cause mortality**	5.35	3.28	7.42	7.17	5.13	9.22
0.1	**Simple multimorbidity (2nd disease)**	2.13	1.27	3.00	2.84	1.99	3.69
0.2	**Third disease**	4.09	2.91	5.28	5.04	3.87	6.21
0.3	**Complex multimorbidity (4th disease)**	5.76	4.13	7.38	7.16	5.56	8.77
1	**Infectious diseases**	4.75	3.18	6.32	5.55	4.00	7.09
2	Bacterial infections	6.43	4.39	8.47	7.27	5.27	9.28
3	Viral infections	-0.76	-5.52	4.01	0.61	-4.06	5.27
4	**Cancers**	2.40	1.10	3.70	0.24	-1.04	1.52
5	Colorectal cancer	3.84	-0.69	8.37	1.63	-2.84	6.11
6	Lung cancer	7.10	1.97	12.23	7.34	2.23	12.45
7	Melanoma	1.36	-1.10	3.82	-1.77	-4.19	0.65
8	Breast cancer (Female)	3.21	0.44	5.97	0.86	-1.91	3.63
9	Prostate cancer (Male)	0.63	-3.39	4.64	-1.75	-5.61	2.11
10	Kidney cancer	5.95	-3.20	15.10	3.42	-5.62	12.45
11	Brain cancer	4.21	-7.05	15.47	-2.21	-13.33	8.90
12	Leukaemia / Lymphoma	3.67	0.02	7.33	-0.48	-4.08	3.13
13	**Blood diseases**	5.07	3.33	6.82	6.69	4.98	8.40
14	Anaemia	4.78	2.89	6.66	6.97	5.11	8.82
15	**Endocrine diseases**	2.52	1.52	3.52	4.32	3.33	5.30
16	Diabetes mellitus	6.75	4.20	9.29	11.42	8.95	13.89
17	**Mental and behavioural disorders**	3.93	2.61	5.26	5.45	4.16	6.75
18	Dementia	1.20	-3.30	5.70	7.43	2.96	11.89
19	Substance use disorders	4.17	2.17	6.17	6.60	4.65	8.55
20	Psychotic disorders	7.46	-3.04	17.96	14.05	3.77	24.33
21	Mood disorders	5.15	2.92	7.38	5.39	3.20	7.58
22	Neurotic disorders	4.37	2.09	6.64	4.86	2.62	7.11
23	**Neurological disorders**	0.94	-0.56	2.44	2.54	1.07	4.01
24	Parkinson disease	2.52	-4.30	9.35	3.15	-3.58	9.88
25	Multiple sclerosis	9.07	-0.19	18.32	6.21	-2.87	15.29
26	Epilepsy	3.46	-2.06	8.99	7.64	2.25	13.03
27	Headaches	1.30	-2.68	5.29	0.66	-3.29	4.61
28	Transient ischaemic attack	0.30	-5.70	6.29	4.03	-1.85	9.91
29	Sleep disorders	2.44	-2.76	7.65	0.45	-4.65	5.56
30	**Eye diseases**	1.58	0.33	2.84	2.17	0.93	3.42
31	**Ear diseases**	0.53	-6.23	7.29	2.96	-3.65	9.56
32	**Circulatory diseases**	1.58	0.80	2.37	2.16	1.39	2.93
33	Hypertension	2.22	1.19	3.25	4.80	3.79	5.81
34	Ischaemic heart disease	1.57	-0.37	3.50	5.14	3.25	7.02
35	*Angina pectoris*	0.68	-2.15	3.51	6.70	3.96	9.44
36	*Myocardial infarction*	0.19	-3.54	3.91	6.41	2.81	10.00
37	Pulmonary embolism	6.52	1.97	11.06	-0.58	-5.13	3.98
38	Arrhythmias	4.25	2.20	6.30	-3.74	-5.78	-1.69
39	Heart failure	5.99	2.50	9.48	4.55	1.09	8.01
40	Cerebrovascular diseases	4.56	1.72	7.41	7.33	4.53	10.13
41	Stroke	3.03	-0.67	6.73	4.34	0.69	7.98
42	*Intracerebral haemorrhage*	2.87	-3.59	9.34	3.49	-2.88	9.86
43	*Cerebral infarction*	3.36	-1.00	7.72	4.80	0.50	9.09
44	Arteriosclerosis	5.73	-1.91	13.37	13.10	5.65	20.55
45	Deep vein thrombosis	4.39	-0.36	9.14	-1.83	-6.55	2.90
46	**Respiratory diseases**	5.62	4.44	6.79	6.41	5.26	7.57
47	Respiratory tract infections	8.97	6.54	11.39	10.11	7.72	12.50
48	Chronic obstructive pulmonary disease	11.11	8.32	13.90	14.70	11.94	17.46
49	Asthma	5.93	3.92	7.94	6.86	4.89	8.82
50	**Digestive system diseases**	2.55	1.81	3.30	2.89	2.16	3.62
51	Appendicitis	0.17	-6.84	7.18	-4.21	-11.04	2.61
52	Inflammatory bowel disease	4.34	1.61	7.07	5.66	2.99	8.33
53	Liver diseases	6.77	3.21	10.33	9.55	6.07	13.03
54	Alcohol-related liver diseases	19.25	7.64	30.85	30.04	18.99	41.09
55	Pancreatitis	10.10	2.30	17.90	11.02	3.36	18.68
56	**Skin diseases**	2.74	1.14	4.35	1.61	0.03	3.19
57	Eczema and skin infections	3.61	0.81	6.40	4.10	1.36	6.84

58	**Musculoskeletal diseases**	0.00	-0.86	0.85	0.11	-0.72	0.95
59	Rheumatoid arthritis	2.68	1.23	4.13	4.12	2.68	5.56
60	Gout	2.19	-2.81	7.19	3.33	-1.51	8.17
61	Osteoarthritis	-2.30	-3.61	-0.98	-2.80	-4.10	-1.50
62	Sciatica	-0.81	-4.01	2.39	-2.21	-5.36	0.94
63	Backpain	1.10	-1.34	3.53	2.00	-0.40	4.39
64	Soft tissue diseases	-0.13	-1.86	1.61	0.04	-1.66	1.74
65	**Genitourinary diseases**	3.60	2.48	4.72	3.11	2.02	4.21
66	Renal failure	4.18	1.98	6.37	4.59	2.42	6.76
67	**Circulatory and respiratory symptoms**	2.60	1.16	4.04	2.73	1.31	4.14
68	**Digestive and abdominal symptoms**	2.58	1.32	3.85	3.36	2.12	4.59
69	**Injury**	3.24	1.63	4.85	2.77	1.19	4.34
70	**Poisoning**	2.65	-3.91	9.20	6.17	-0.22	12.55
71	**Road accidents**	-2.58	-7.55	2.38	-4.78	-9.52	-0.04
72	**Fall**	5.15	3.17	7.13	4.65	2.69	6.61
73	**Self-harm**	0.12	-8.31	8.54	4.61	-3.52	12.75

Supplement eTable 8. Associations of obesity with outcomes with minimal adjustment

	HR for obesity vs normal weight defined using		
**Outcome**	**BMI**	**WC**	**WHtR**
**UK Biobank**			
All-cause mortality	1.65 (1.60-1.70)	1.66 (1.61-1.71)	1.66 (1.61-1.71)
Simple multimorbidity (2nd disease)	2.99 (2.95-3.04)	3.00 (2.95-3.04)	3.00 (2.95-3.05)
Third disease	3.72 (3.65-3.80)	3.73 (3.66-3.81)	3.74 (3.66-3.81)
Complex multimorbidity (4th disease)	4.58 (4.46-4.70)	4.60 (4.48-4.72)	4.60 (4.48-4.72)
**Whitehall**			
All-cause mortality	1.81 (1.59-2.04)	1.82 (1.61-2.06)	1.92 (1.70-2.17)
Simple multimorbidity (2nd disease)	2.50 (2.27-2.75)	2.61 (2.37-2.87)	2.60 (2.36-2.86)
Third disease	2.68 (2.39-3.00)	2.78 (2.49-3.11)	2.77 (2.47-3.10)
Complex multimorbidity (4th disease)	3.36 (2.95-3.83)	3.38 (2.97-3.85)	3.42 (3.00-3.90)

Numbers presented are HR (95% CI) compared with the normal weight group and its WC/WHtR equivalent.
Adjusted for age, sex, and ethnicity.

Supplement eTable 9. Difference in population attributable fractions between obesity defined using different measures with minimal adjustment

	**Obesity (BMI ≥ 30)**						**Overweight (BMI ≥ 25 & < 30)**					
	**PAF_{WC} - PAF_{BMI}**			**PAF_{WHtR} - PAF_{BMI}**			**PAF_{WC} - PAF_{BMI}**			**PAFWHtR - PAFBMI**		
**Outcome**	**%**	**Lower CI**	**Upper CI**	**%**	**Lower CI**	**Upper CI**	**%**	**Lower CI**	**Upper CI**		**Lower CI**	**Upper CI**
**UK Biobank**												
All-cause mortality	0.21	-1.79	2.21	0.22	-1.78	2.22	7.80	5.71	9.88	10.62	8.58	12.66
Simple multimorbidity (2nd disease)	0.07	-0.73	0.86	0.07	-0.72	0.87	2.51	1.66	3.37	3.12	2.28	3.96
Third disease	0.13	-0.82	1.08	0.14	-0.81	1.10	4.68	3.51	5.86	5.67	4.52	6.83
Complex multimorbidity (4th disease)	0.18	-0.94	1.29	0.19	-0.92	1.31	6.31	4.71	7.90	7.78	6.21	9.36
**Whitehall**												
All-cause mortality	-0.19	-1.41	0.91	0.75	-0.56	2.03	4.27	1.49	7.43	2.67	-0.23	5.60
Simple multimorbidity (2nd disease)	0.47	-0.90	1.94	0.60	-0.90	2.00	1.86	-0.33	3.72	0.72	-1.32	2.86
Third disease	0.34	-1.34	1.87	0.38	-1.19	1.88	1.92	-0.93	4.46	1.49	-1.17	3.99
Complex multimorbidity (4th disease)	-0.23	-2.45	1.40	0.02	-2.01	1.81	1.81	-1.74	4.73	2.02	-1.11	5.24

PAF assumes the HR estimated to be causal which may not be true.

HRs were adjusted for age, sex, and ethnicity.

eFigure 1 Associations of adiposity markers with infections and cancer outcomes

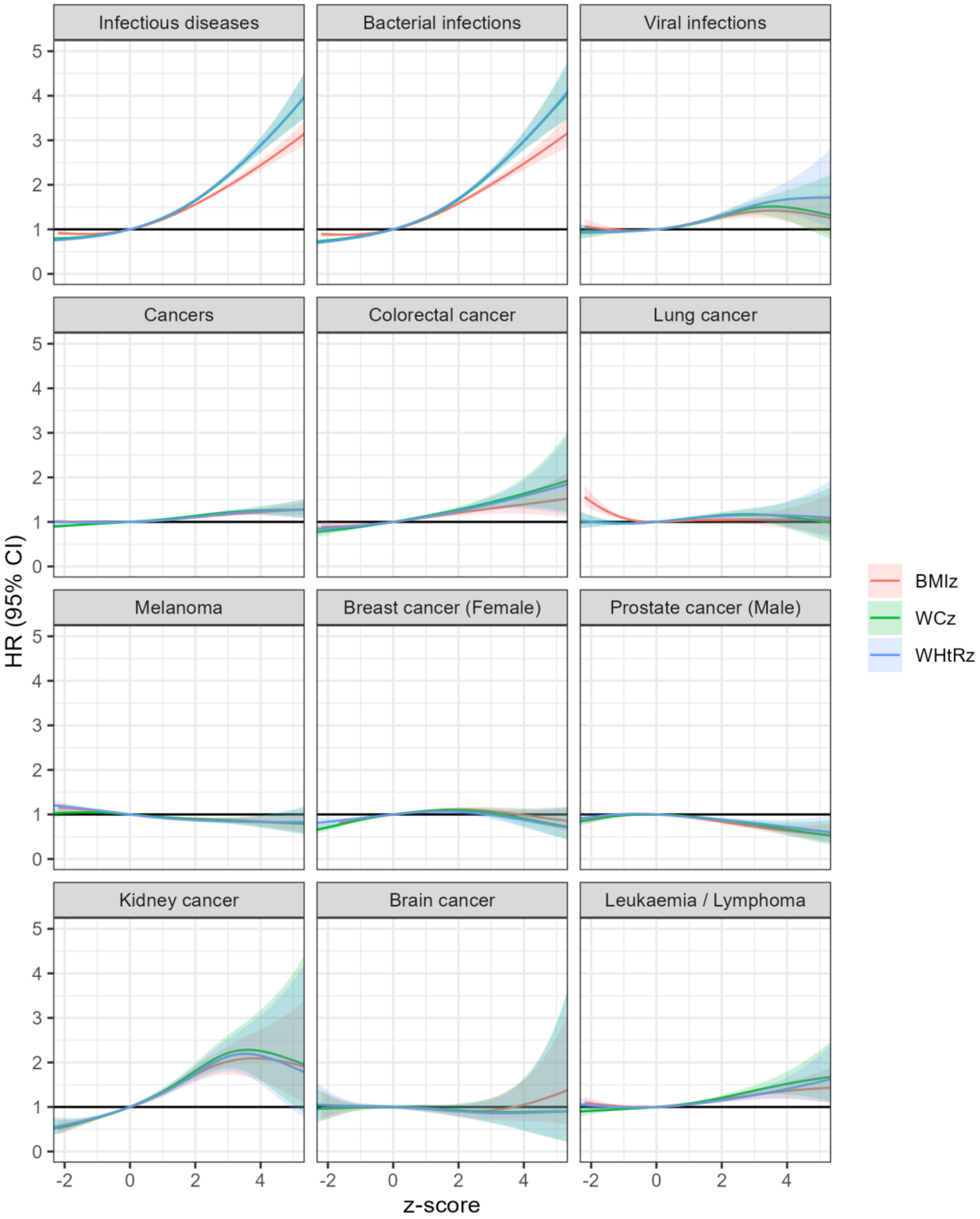

eFigure 2 Associations of adiposity markers with blood and endocrine and mental health outcomes

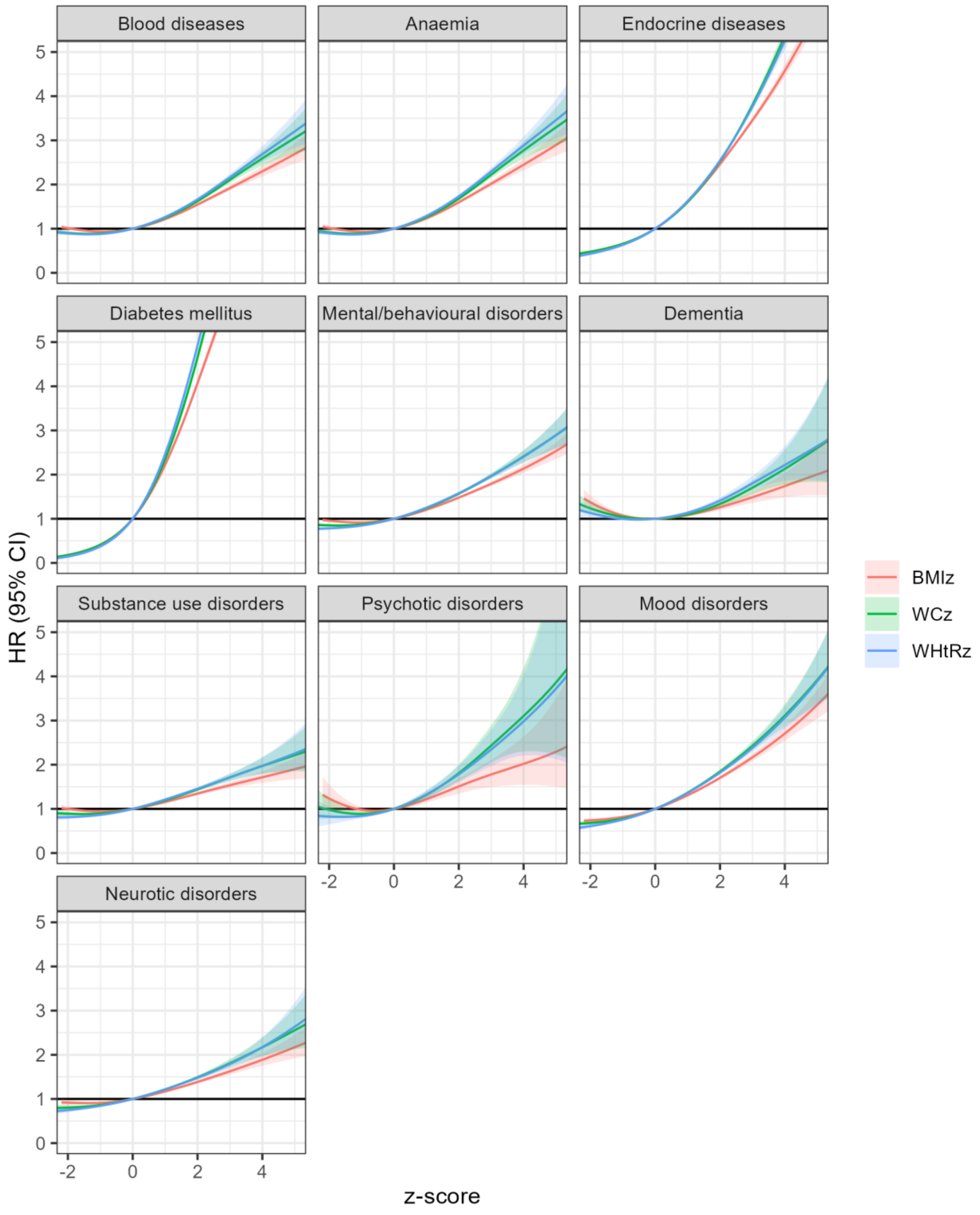

eFigure 3 Associations of adiposity markers with neuro and eye and ear outcomes

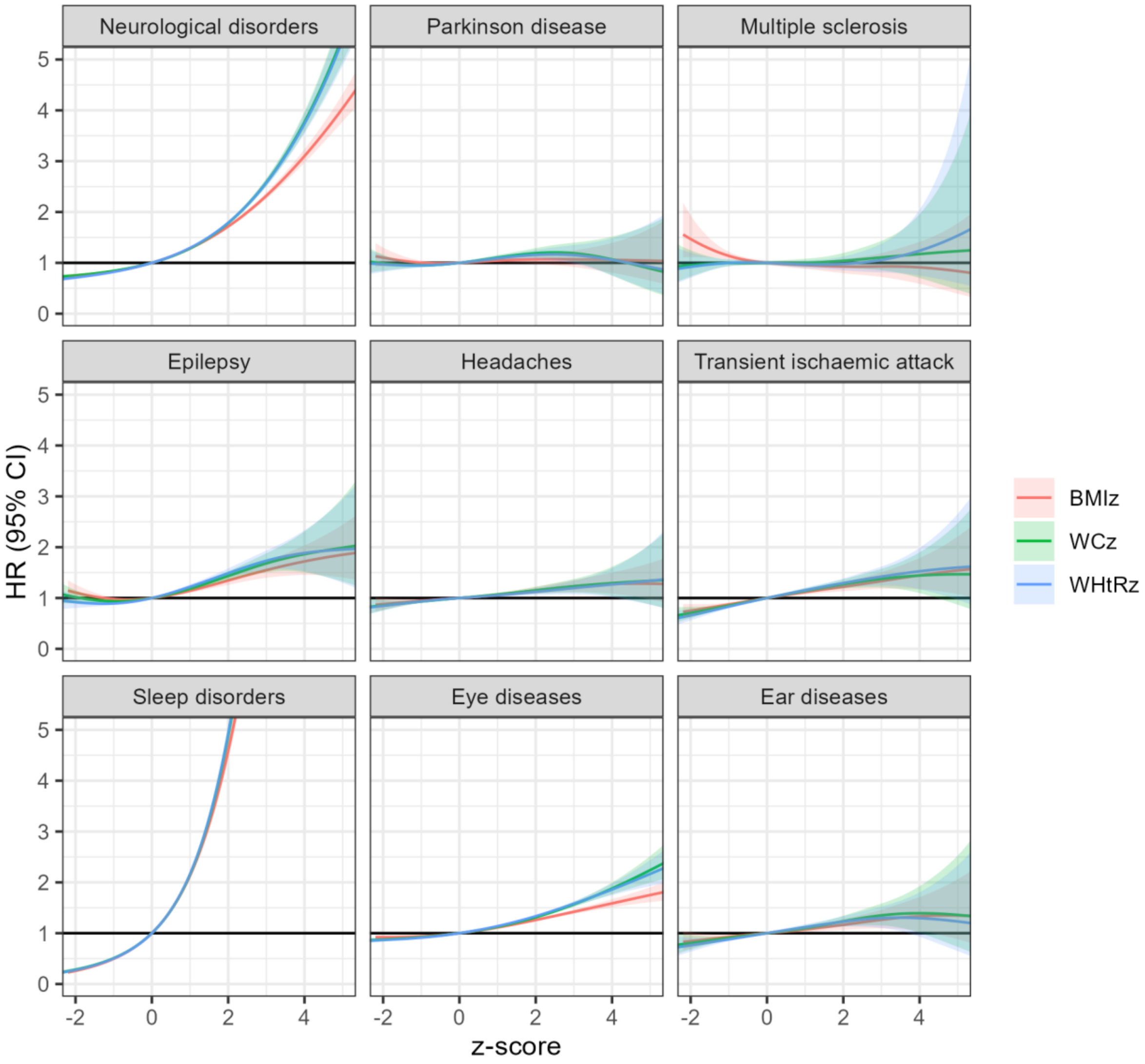

eFigure 4 Associations of adiposity markers with circulatory outcomes

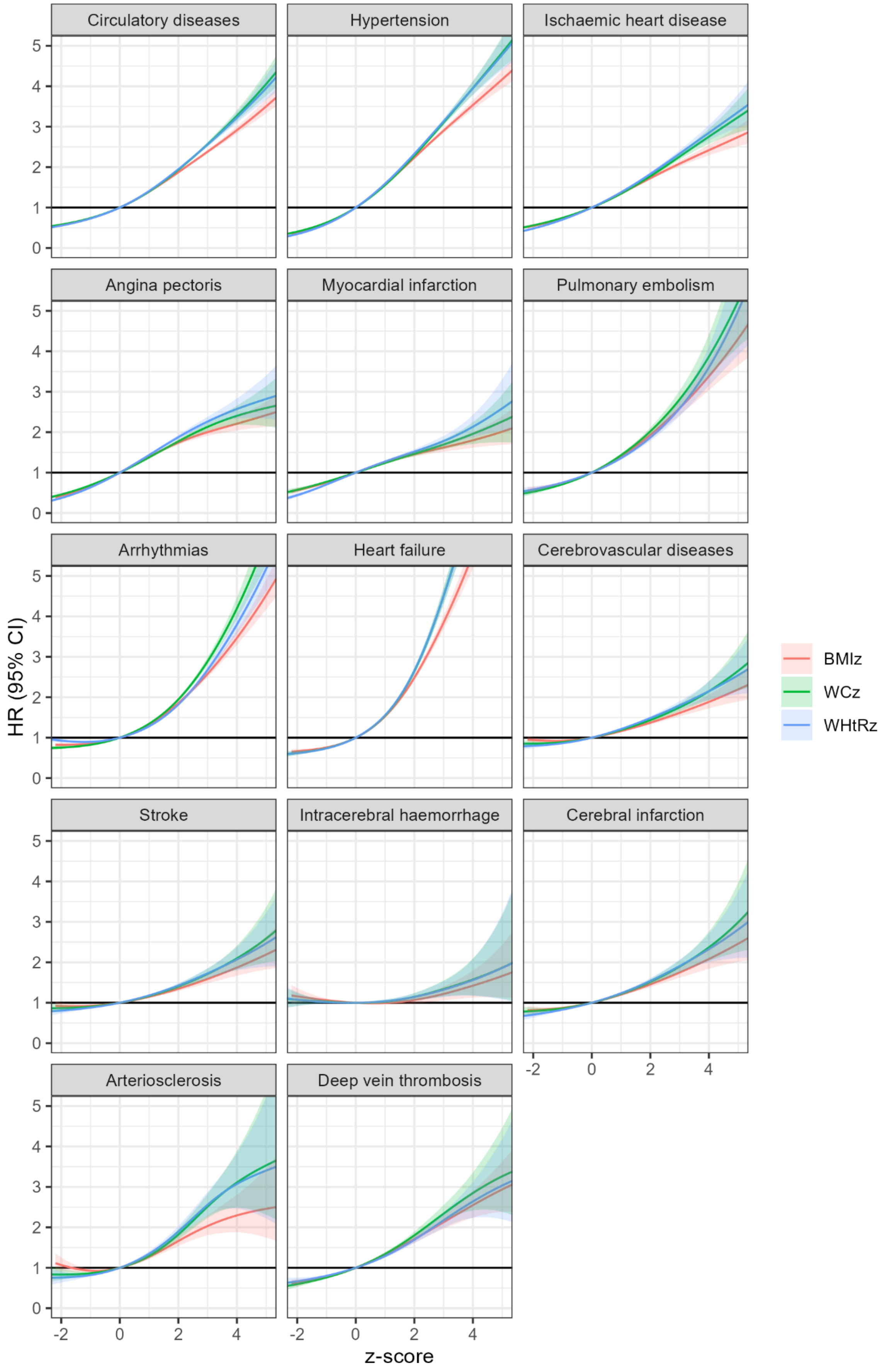

eFigure 5 Associations of adiposity markers with respiratory and digestive outcomes

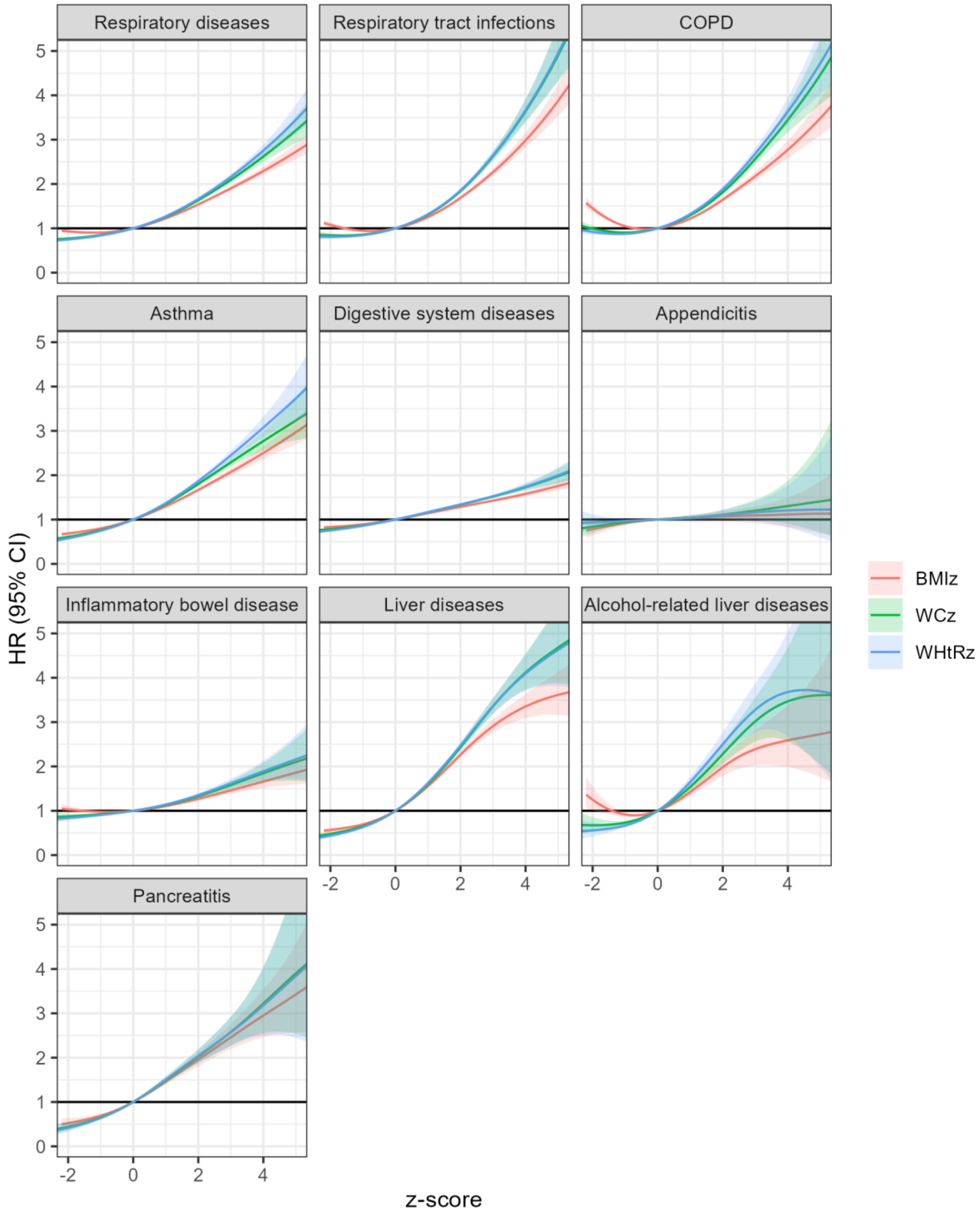

eFigure 6 Associations of adiposity markers with skin and musculoskeletal outcomes

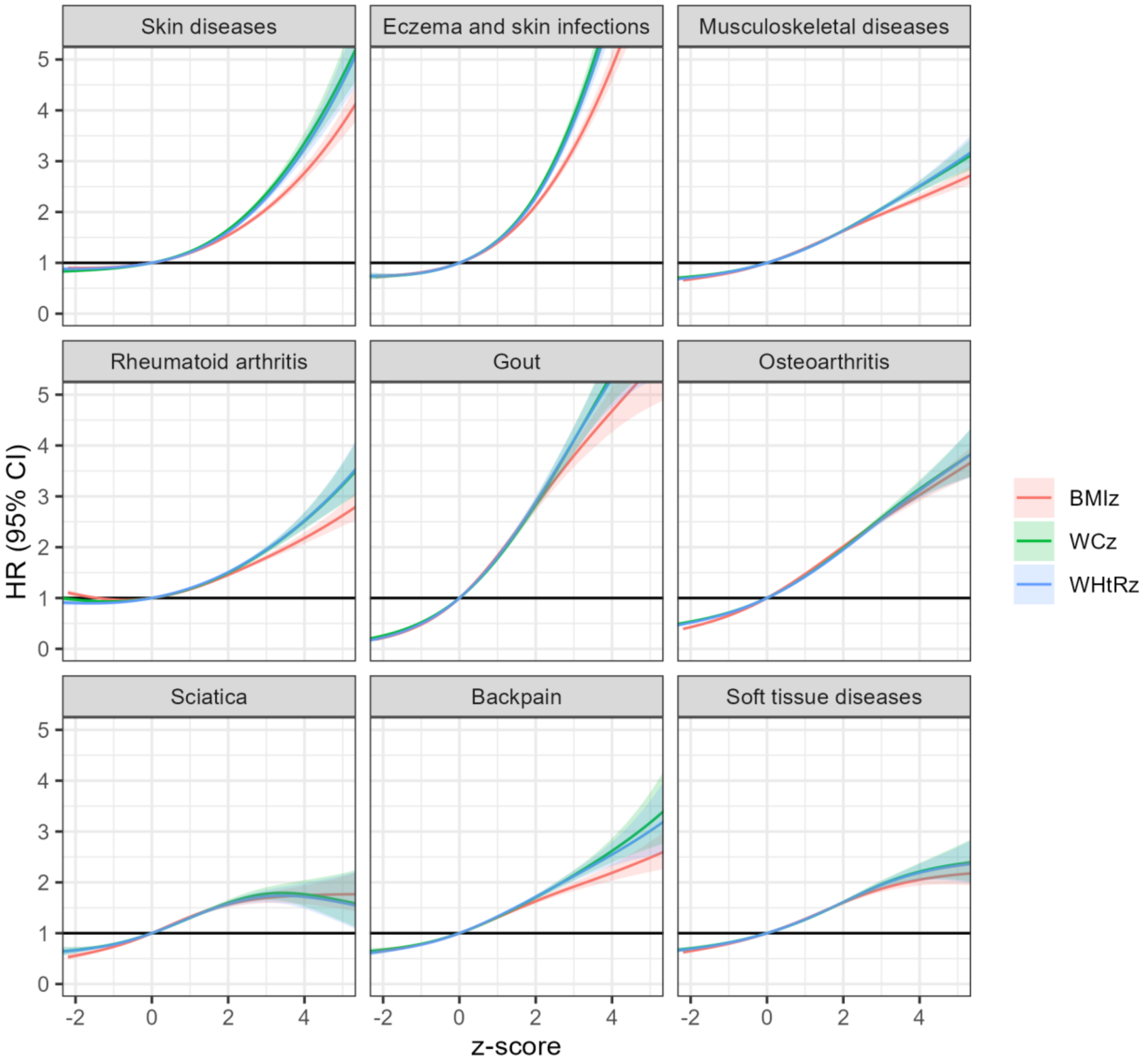

eFigure 7 Associations of adiposity markers with genitourinary and miscellaneous outcomes

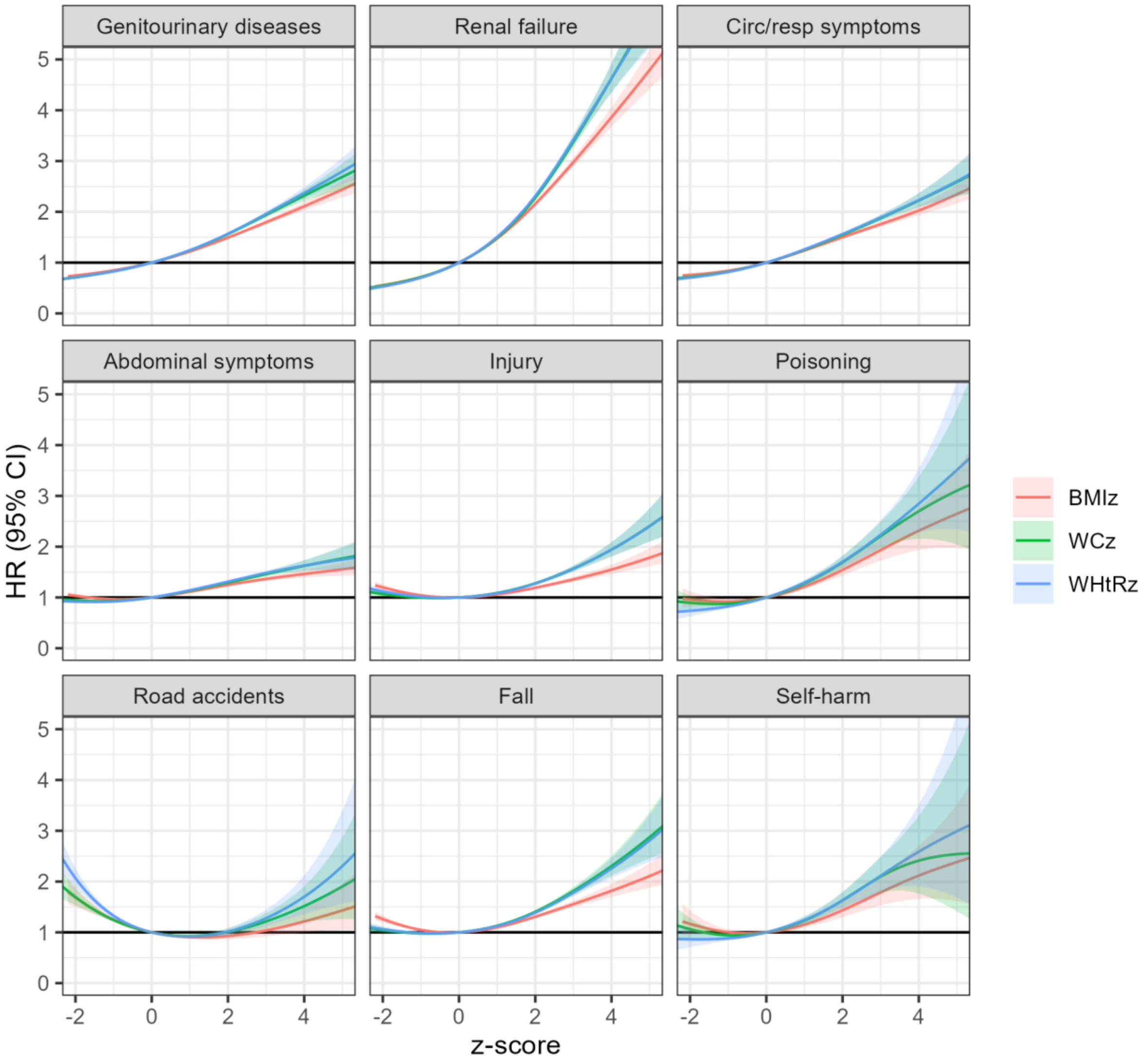

Supplement: Supplement 1 [file media-1.pdf]
